# Nanomolar Protein Thermal Profiling with Modified Cyanine Dyes

**DOI:** 10.1021/acs.analchem.3c02844

**Published:** 2023-12-07

**Authors:** Morteza Malakoutikhah, Randa Mahran, Negin Gooran, Ahmadreza Masoumi, Katri Lundell, Arto Liljeblad, Keelan Guiley, Shizhong Dai, Qinheng Zheng, Lawrence Zhu, Kevan M. Shokat, Kari Kopra, Harri Härmä

**Affiliations:** † Department of Chemistry, University of Turku, Henrikinkatu 2, 20500 Turku, Finland; ‡ Laboratory of Synthetic Drug Chemistry, Institute of Biomedicine, University of Turku, Kiinamyllynkatu 10, FI-20520 Turku, Finland; § Department of Cellular and Molecular Pharmacology and Howard Hughes Medical Institute, University of California, San Francisco, California 94158, United States; ∥ Current address: Rezo Therapeutics, Inc., San Francisco, California 94158, United States; ⊥ Current address: Department of Genetics, Stanford University, Stanford, California 94305, United States

## Abstract

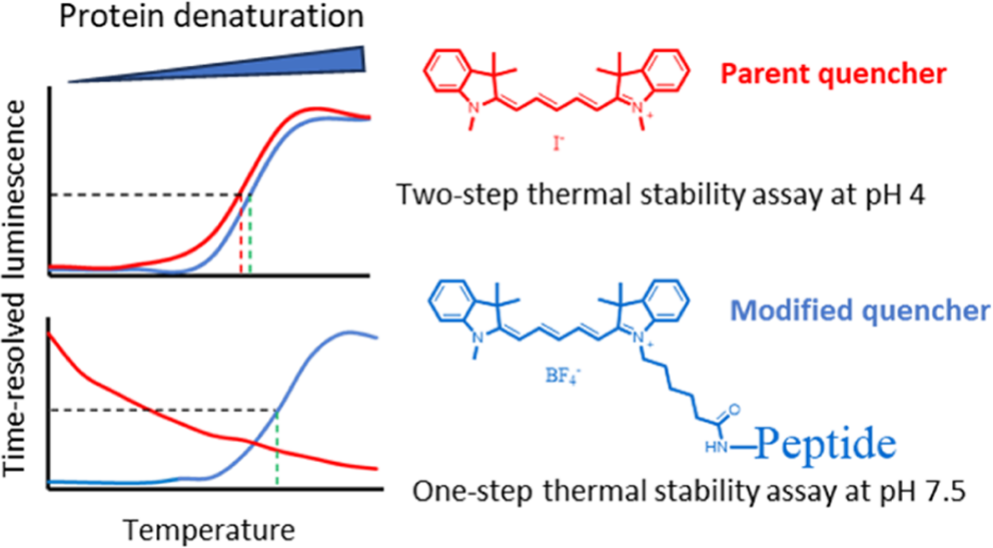

Protein properties and interactions have been widely investigated by using external labels. However, the micromolar sensitivity of the current dyes limits their applicability due to the high material consumption and assay cost. In response to this challenge, we synthesized a series of cyanine5 (Cy5) dye-based quencher molecules to develop an external dye technique to probe proteins at the nanomolar protein level in a high-throughput one-step assay format. Several families of Cy5 dye-based quenchers with ring and/or side-chain modifications were designed and synthesized by introducing organic small molecules or peptides. Our results showed that steric hindrance and electrostatic interactions are more important than hydrophobicity in the interaction between the luminescent negatively charged europium-chelate-labeled peptide (Eu-probe) and the quencher molecules. The presence of substituents on the quencher indolenine rings reduces their quenching property, whereas the increased positive charge on the indolenine side chain improved the interaction between the quenchers and the luminescent compound. The designed quencher structures entirely altered the dynamics of the Eu-probe (protein-probe) for studying protein stability and interactions, as we were able to reduce the quencher concentration 100-fold. Moreover, the new quencher molecules allowed us to conduct the experiments using neutral buffer conditions, known as the peptide-probe assay. These improvements enabled us to apply the method in a one-step format for nanomolar protein–ligand interaction and protein profiling studies instead of the previously developed two-step protocol. These improvements provide a faster and simpler method with lower material consumption.

## Introduction

Proteins are essential biopolymers that play crucial roles in various biochemical processes. An increasing number of therapeutic proteins have been developed; for example, during 2014 and 2018, 116 therapeutic proteins were approved in the United States and European Union.^1^ The stability of these proteins is a vital factor in their correct function. Different environmental conditions, such as pH,^2,3^ temperature,^4^ ionic strength,^3,5^ the presence of organic molecules,^6^ or other proteins,^7^ and mechanical agitation,^8^ can affect protein integrity and subsequently their stability and activity. Due to the significance of protein stability, numerous methods have been developed to monitor and determine structural changes in proteins. These methods can be categorized into two main categories: label-based and label-free techniques.

Label-based techniques require a detectable label, such as a fluorophore, to be incorporated into proteins prior to analysis.^9,10^ However, this labeling may affect the protein structure, activity, and stability. To overcome these obstacles, label-free methods such as differential scanning calorimetry (DSC),^11,12^ circular dichroism (CD),^13^ UV spectroscopy,^14^ and fluorescence-based assays have gained more attention.^15^

One such fluorescence-based method is differential scanning fluorimetry (DSF), in which a change in fluorescence intensity is monitored due to changes in the environment of the reporter molecule. Intrinsic fluorescence is due to the presence of aromatic amino acids, primarily tryptophan residues, in the protein sequence. Protein unfolding alters the environment of tryptophan by exposing it to solvent, potentially affecting both the monitored fluorescence intensity and spectra. Extrinsic fluorescence is provided by the addition of an environmentally sensitive fluorescent dye such as SYPRO Orange and 1-anilinonaphthalene-8-sulfonic acid (ANS). In the native state, there is minimal interaction between the protein and the fluorescent dye, and thus, the fluorescence intensity of the dye is significantly quenched by water. However, protein denaturation exposes hydrophobic regions of the protein, leading to increased dye binding and protection from water, thus enhancing the observed fluorescence intensity.^15^

It is worth mentioning that fluorescence-based methods can also be used to investigate protein–ligand interaction (PLI) and protein–protein interaction (PPI) because the presence of an interacting ligand or protein can alter the stability of the protein of interest.^16−20^ Although DSF has been widely used to study protein stability and PLI,^15−17,21−24^ it requires micromolar protein concentration,^17,24^ potentially promoting protein aggregation and leading to high background signals in the presence of native proteins.

**Figure 1:**
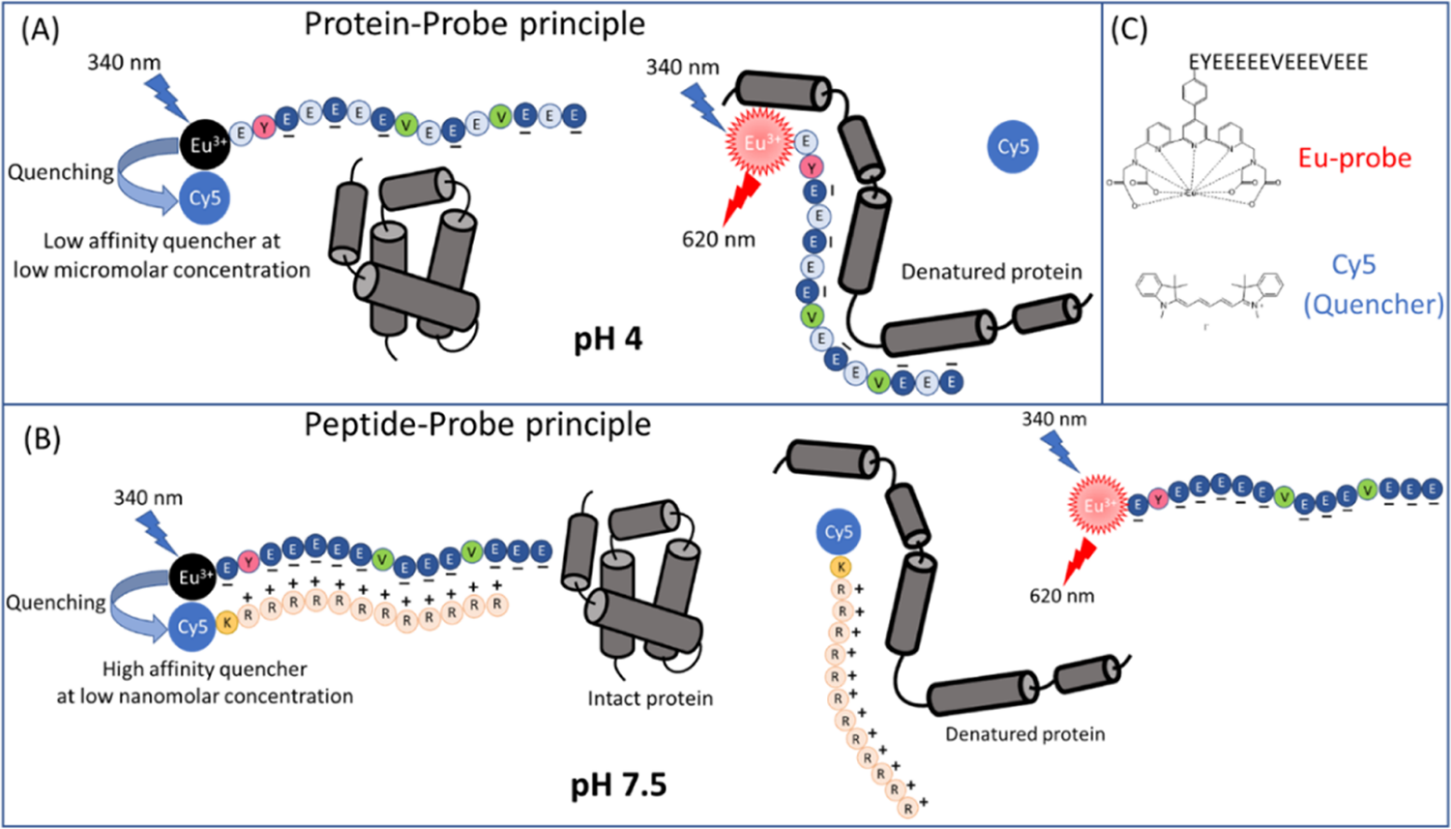
(A) Protein-probe principle: intact protein has minimal interaction with the Eu-probe, and the presence of Cy5 quenches the TRL-signal of the probe. When the protein is thermally denatured, the Eu-probe binds to the exposed hydrophobic regions. As a result, the interaction between Cy5 and the Eu-probe is reduced, leading to a high TRL-signal measurement. (B) The peptide-probe principle: the modified and synthesized quencher, known as a peptide quencher, exhibits a high affinity with the Eu-probe, resulting in efficient quenching of the TRL-signal. Thermally denatured protein disrupts the interaction between negatively and positively charged peptides, leading to a high TRL-signal. (C) Chemical structures of the Eu-probe and Cy5 (quencher).

To enhance the sensitivity and simplicity of protein stability and PLI assays, we have developed the protein-probe technique, which utilizes time-resolved luminescence (TRL) detection without the need for protein labeling.^25^ This method employs a luminescent europium(III) chelate-labeled peptide (Eu-probe) and a soluble quencher molecule (Figure 1). The protein-probe is a two-step assay in which the protein of interest is first thermally denatured, and then a modulation solution containing the sensing elements is added (Figure S1). As with other DSF-type methods, thermal denaturation exposes protein inner regions and promotes the binding of the Eu-probe, which can be monitored with an increase in TRL-signal (Figure 1). We have demonstrated that the protein-probe method is highly sensitive and can detect protein interactions, stability, aggregation, and enzyme activities with 100-fold higher sensitivity than the commonly used SYPRO Orange.^7,25−28^ In this study, we describe our scientific efforts to further improve the sensitivity and simplicity of the assay by modifying the quencher structure; 13 quencher molecules were prepared and studied using the protein-probe platform. Our results demonstrate significant improvements in assay performance, enabling the detection of protein stability and protein–ligand interaction using a one-step peptide-probe protocol at neutral pH buffers (Figure 1).

## Experimental Section

### Materials and Methods

The experimental materials, methods, and instrumentation details are provided in the Supporting Information.

#### Two-Step Protein-Probe

Unless otherwise specified, the assays were performed in an 8 μL sample volume (0.1× PBS), with 2–3 replicates. Then, the protein-probe (modulation) solution, containing phosphate–citrate buffer (7.7 mM Na_2_HPO_4_, 6.1 mM citric acid, pH 4) supplemented with 0.01% Triton X-100, quencher (1 μM), and Eu-probe (1 nM), was added in 65 μL volume.

In the first step, 8 μL of a protein sample (300 nM in 0.1× PBS pH 7.5) was added to a 96-well plate and heated at 85 °C for 3 min to denature the protein, and then in the second step, 65 μL of the modulation solution was added and TRL-signal was measured. TRL-signal from the thermally denatured protein is divided by that from the native protein to give the signal-to-background (S/B) ratio.

#### One-Step Peptide-Probe Using 384-Well Plates

Unless otherwise specified, all assays were performed in a 20 μL total volume, which includes 5 μL of target protein (1.2 μM) or protein + inhibitors (1.2 + 15 μM) and 15 μL of the modulation solution (Q14 10 nM and Eu^3+^-probe 1 nM). Both target proteins and detection solution were prepared in 10 mM HEPES pH 7.5, 10 mM NaCl, 0.01% Brij 30 buffer and added to a 384-well 4titude PCR plate. The mixture was then heated at the desired temperatures (ranging 30–95 °C with a temperature step interval of 5 °C) and TRL-signal was immediately measured after each temperature. Protein concentrations for the peptide-probe assay varied from 100 to 500 nM.

### Eu-Probe and Quencher Preparation

Their preparation is detailed in the Supporting Information.

## Results and Discussion

In this study, we have developed a one-step method for protein thermal profiling at nanomolar protein concentrations (Figure S1). Our previous work was based on a peptide bound to Eu-chelate (Eu-probe) and a Cy5 dye quencher (1,1,3,3,3′,3′-hexamethylindodicarbocyanine iodide) (Figure 1), which provided a two-step procedure for studying protein denaturation and measuring protein melting temperature using phosphate-citrate buffer (pH 4) as the detection solution.^25^ However, this two-step protein-probe method requires a fresh, intact protein sample for each tested temperature, which increases the material consumption, leading to a somewhat cumbersome thermal profiling process. Although the method is highly flexible, as any temperature can be selected in any order, reduced material consumption is not achieved, and the assay is difficult to automate. Therefore, in this study, we focused on developing the quencher structure to understand the interactions between the Eu-probe and the quencher and to establish a one-step profiling protocol at neutral pH (Figure S1).

### Cy5 Quencher Synthesis and Characterization

**Figure 2:**
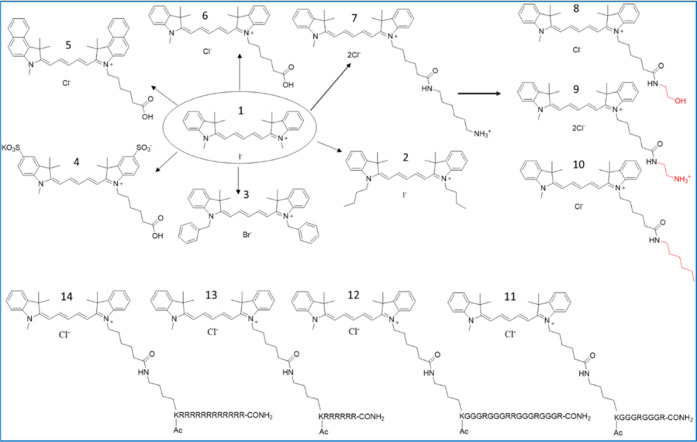
Structures of Cy5 dye quenchers **1**–**14** (Q1–Q14).

The quencher previously used, referred to as parent quencher (Q1) in this study, is a symmetric Cy5 dye with two indolenine rings and a delocalized positive charge (Figure 1C).^25^ Q1 served as a basis for modifying and studying the quencher properties. To achieve this, we prepared quenchers **2**–**7** (Q2–Q7) that differ only in the substituents on the indolenine groups (Figure 2). We synthesized hydrophobic symmetric quenchers Q2–Q3 using a microwave-assisted approach with purity greater than 90% (Figures S2 and S3).^29−32^ Commercially available quenchers Q4–Q7 were purchased.

First, we evaluated the quenching ability of the newly synthesized quenchers (1 μM) with the Eu-probe (1 nM) by mixing the components in the phosphate-citrate buffer, pH 4. Among the quencher library, Q1 showed greater quenching efficiency than Q2–Q5 and similar to that of Q6 (Figure S4). However, Q7 displayed significantly improved quenching properties compared to Q1. Hence, we concluded that the electrostatic interaction between the positively charged side-chain amine (Q7) and the negatively charged peptide of the Eu-probe is critical to improving the interaction and assay performance.

Next, to assess the functionality of the quenchers in the assay, we measured a monoclonal antibody as a model protein under both native and denatured conditions. This was accomplished through a two-step protocol where the antibody was heated for 3 min at 85 °C before the addition of the detection solution.^25^ The results demonstrated that Q7, with the highest quenching ability, produced the greatest S/B ratio (14.5) in the denaturation assay. This ratio was approximately 3 times higher than that of Q1, which gave an S/B ratio of 5.4 under the same conditions (Figure S5). Q6 had an S/B ratio of 2.6, despite its quenching ability being similar to that of Q1. Quenchers Q2–Q5 resulted in very low S/B ratios due to their low quenching ability at the given concentration (1 μM) and buffer conditions. Interestingly, at high quencher concentration (up to 8 μM), Q7 still displayed the highest S/B ratio among all tested quenchers (data not shown).

As Q7 containing amide and amine functional groups on its indolenine side chain outperformed Q1 in terms of quenching and assay properties, a question arose regarding whether these improvements were due to the amide bond, the amino group, or the hexyl group in the indolenine side chain. To answer this question, we labeled three different amine-containing molecules with Cy5 NHS ester to prepare quenchers **8–10** (Q8–Q10) (Figures 2 and S6–S8). This resulted in three compounds containing an amide bond with a hydrophilic hydroxyl group (Q8), an amine group with an alkyl chain shorter than that of quencher **7** (Q9), and a hydrophobic hexyl group containing quencher (Q10). The performance of Q8–Q10 was evaluated in the protein-probe antibody denaturation assay alongside Q1 and Q7 as controls. Notably, Q7 exhibited the highest S/B ratio (thermally induced denatured vs intact) among all of the tested quenchers (Figure S5).

To further validate the previous findings and improve the assay sensitivity, four different peptide sequences were labeled with Cy5 NHS ester to produce quenchers **11**–**14** (Q11–Q14), which had varying numbers of positive amino groups (Figures 2 and S9–S12). In the protein-probe antibody denaturation assay, novel Q11–Q14 exhibited a significant increase in the S/B ratio, as depicted in Figure S13. Q14 showed a remarkable 9-fold higher S/B ratio (43.6) compared to Q1 (4.6). While hydrophobicity is known to govern the interaction between cyanine dyes and proteins such as serum albumin,^33^ this was not the case for Q11–Q14 and the Eu-probe. Interestingly, we observed a clear reverse correlation between the relative hydrophobicity of Q11–Q14, as determined by their retention times on a reversed-phase HPLC column (Figures S9–S12) and their affinities for the Eu-probe. These results indicate that regardless of the peptide sequence in the structure of the quenchers, there is a correlation between the number of positively charged amino acids in the peptide sequence and the S/B ratio, likely due to the strong interaction between the negatively charged Eu-probe and positively charged quencher peptides.

To investigate this interaction further, we mixed a constant concentration of the Eu-probe (1 nM) with different concentrations (1–1000 nM) of Q1, Q7, and Q13 in water (pH 7) and phosphate–citrate buffer (pH 3.4) containing 0.01% Triton X-100 and measured the TRL-signal of the resulting mixtures (Figure S14). An increase in the quencher concentration led to reduced TRL-signal values for these three quenchers at pH **7**, with the most significant reduction observed for Q13 compared to that for Q1 and Q7 (Figure S14A). This implies that Q13 has a strong interaction with the Eu-probe due to its high number of positive charges. The results of the same experiments at phosphate–citrate buffer pH 3.4 further demonstrate the importance of the charge-based peptide interaction, as this low pH neutralizes most of the negatively charged Eu-probe (Figure S14B). At pH 3.4, Q13 had an effect on TRL-signals only at high nanomolar concentration (>100 nM), while at pH 7, almost maximal TRL-signal quenching was achieved at low nanomolar concentrations (10 nM) (Figure S14). Q1 and Q7 exhibited similar behavior to Q13, albeit with a lower quenching efficiency. This can be explained by the fact that at pH 7, the side-chain carboxylate (p*K*_a_ = 4.15) of the Eu-probe glutamic acid residues is unprotonated and negatively charged, while at pH 3.4, the residues are mostly protonated, resulting in relatively weak interaction between Q13 and the Eu-probe. We also performed experiments with the Eu-chelate alone, lacking the negatively charged peptide, instead of the Eu-probe and observed no difference in the quenching ability of the three quenchers in water (pH 7) and the phosphate–citrate buffer (pH 3.4) (Figure S15). These findings support our hypothesis for the interaction of positively and negatively charged peptides of Q13 and the Eu-probe, respectively.

When the protein-probe assay was performed with Q13 (1 μM) in different buffers, phosphate–citrate buffers with different pH values (4, 5, 6, 7), HEPES (10 mM, pH 7.5), and Tris (10 mM, pH 7.5), it exhibited a higher S/B ratio at acidic pH ranges (4 and 5) than at neutral pH in the antibody assay (Figure S16). The likely explanation is that at neutral pH and high concentration of Q13 (1 μM), the strong interaction between the positively charged Q13 and the negatively charged Eu-probe is excessive, preventing the interaction between the Eu-probe and the denatured protein. Therefore, we next estimated the binding strength of the arginine-rich peptide quencher and the parent quencher for the Eu-probe.

To investigate the interaction between highly positively charged Q14 and Q1 with the Eu-probe, we monitored the binding curves for the quenchers. A dilution series of Q1 (0.009–20 μM) and Q14 (0.04–0.1 μM) were assayed with the Eu-probe (1 nM) in H_2_O (pH 7) (Figure S17). The EC_50_ values (half-maximal effective concentration) of Q1 and Q14, calculated from these dilution curves, were 131.0 ± 3.9 and 1.9 ± 0.05 nM, respectively. The 69-fold lower EC_50_ of Q14 compared to that of Q1 suggests that Q14 has significantly greater affinity and, therefore, quenching capacity for the Eu-probe than Q1 under neutral pH conditions, with the Eu-probe remaining negatively charged and Q14 positively charged.

Given the significantly higher binding affinity of Q14 than that of Q1, and the fact that a lower concentration of arginine-rich peptide quencher (10 and 100 nM, Figure S14) provides sufficient quenching efficiency at neutral pH (7) compared to acidic pH (3.4), we expected to find a higher S/B ratio at neutral pH and lower concentrations than 1000 nM for Q14. Therefore, we measured the protein-probe antibody assay using different concentrations of Q14 (1.56–200 nM) in various buffers (Figure S18). We did not compare this to Q1, as according to the binding strength studies, this quencher was not functional at the low concentration range. The results were surprising, as the functionality of the protein-probe antibody assay significantly varied according to the pH and quencher concentration. High S/B ratios were obtained at pH 4 and concentrations above 100 nM. However, an enhanced S/B ratio was observed in the neutral pH range at lower quencher concentrations, approximately 10 nM (peptide-probe). These findings suggest that an improved protein-probe assay can be based on carefully selected quencher structure and assay condition.

The fluorescence spectra for all quenchers were measured, revealing similar excitation and emission patterns for Q2–Q14 to Q1 studied in DMSO and/or H_2_O. Exceptions for excitation and emission spectra were found for Q4 in DMSO and Q5 in both solvents (Figures S19–S26). We also found that the fluorescence spectra of Q14 were not affected by the presence of the Eu-probe or the protein (Figures S27 and S28).

### One-Step Peptide-Probe Assay

The novel peptide-based quenchers improved assay performance in the two-step protocol at acidic pH and enabled us to perform the assay at neutral pH. Their ability to function at neutral pH (peptide-probe, Figure 1) holds the potential for a one-step peptide-probe protocol, offering even greater advantages (Figure S1). Therefore, we studied Q14 for protein thermal stability and PLI using a one-step protocol at pH 7.5. The exact mechanism of the interaction between the denatured protein, the Eu-probe, and Q14 is not known. However, we hypothesize that the denatured protein most likely interacts with the peptide quencher, as it is known that the Eu-probe, which is completely negatively charged at neutral pH (7.5), does not bind to the denatured protein (Figure 1). The one-step peptide-probe protocol significantly reduces the consumption of required proteins and assay components as the entire assay can be run within a single well by simply ramping the temperature (Figure S1B). This makes the method amenable to automation with reduced costs.

To measure melting temperatures (*T*_m_) of proteins with the one-step peptide-probe protocol, we ran the assay with the Eu-probe (1 nM) and Q14 (12.5 nM) in a Tris buffer (10 mM, pH 7.5) supplemented with 0.01% Triton X-100. During the method development, the functionality was proved with an antibody as a model protein. Next, we selected bovine carbonic anhydrase (BCA), a highly applied DSF model protein, to show protein–ligand interaction. We first ran the thermal stability profile of BCA (33 nM) in the absence and presence of a single concentration of acetazolamide (AZA, 0.55 μM) (Figure S29A). AZA is a known BCA stabilizing inhibitor resulting in a well-characterized increase in BCA *T*_m_ value.^14,25^ The *T*_m_ values were 61.9 ± 0.7 and 63.4 ± 0.7 °C for BCA and BCA + AZA, respectively. The observed Δ*T*_m_ with the developed method was 1.5 °C, and the S/B ratios in the absence and presence of AZA were 4.7 and 4.5, respectively. The observed Δ*T*_m_ is similar to the values reported in the literature,^14,25^ indicating the correct function and potential of the one-step peptide-probe assay scheme.

In the study of the two-step protocol and the one-step peptide-probe PLI assay with BCA, the experiments were performed in a 96-well microtiter plate using 8 μL of protein solution and 65 μL of the detection solution (Figure S1A). Since these conditions were directly derived from the original protein-probe protocol,^25^ we then optimized the assay scheme for a 384-well plate format commonly used in high-throughput screening (HTS) processes. To achieve this, we reduced the assay volume to 20 μL (Figure S1B). Additionally, we replaced the previously used Tris buffer with a HEPES buffer to improve signal stability in the new 384-well plate one-step peptide-probe protocol. With these modifications, we remeasured the BCA-AZA PLI assays (Figure S29B) and obtained *T*_m_ values of 64.6 ± 0.3 and 67.5 ± 0.4 °C for BCA (300 nM) and BCA + AZA (300 nM + 3.75 μM), respectively. The observed *T*_m_ values and Δ*T*_m_ (2.9 °C) were slightly higher than those in the 96-well plate protocol, possibly due to differences in assay buffers, plates, volumes, proteins, and AZA concentrations (Table S1).^15,26,34,35^

**Figure 3:**
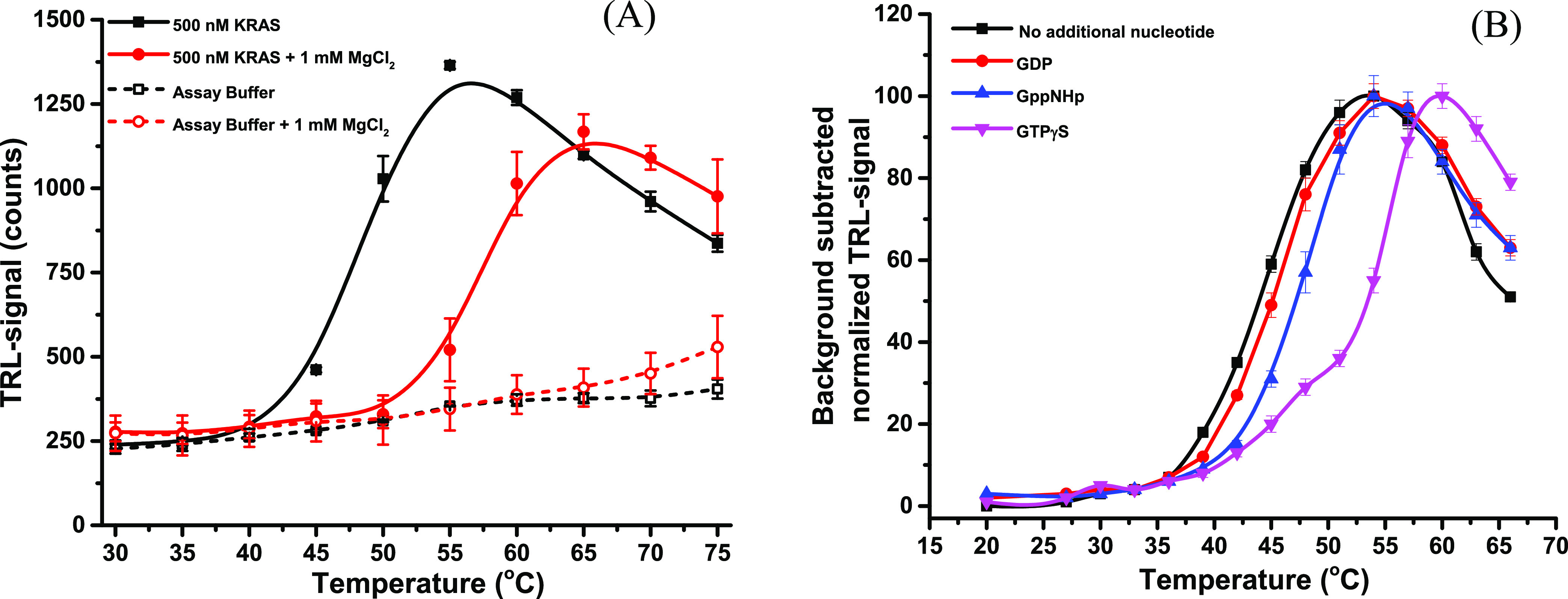
(A) Melting curves of KRAS in both the absence and presence of MgCl_2_ (1 mM) using the one-step peptide-probe assays with the Eu-probe (1 nM) and Q14 (10 nM) in HEPES buffer (10 mM, pH 7.5, 10 mM NaCl, 0.01% Brij 30). The *T*_m_ values were 47.6 ± 0.6 and 56.7 ± 0.5 °C for KRAS and KRAS + MgCl_2_ (1 mM), respectively. (B) Detection of nucleotide exchange using 100 nM GDP-loaded GαS (R201C, C237S) in the absence and presence of additional GDP, GppNHp, and GTPγS (5 μM) with the one-step peptide-probe assays with the Eu-probe (7.5 nM) and Q14 (7.5 nM) at HEPES buffer (10 mM, pH 7.4, 10 mM NaCl, 1 mM MgCl_2_, 0.01% Triton X-100). The *T*_m_ values for GαS (R201C and C237S) alone with GDP, GppNHp, and GTPγS were 44.4 ± 0.2, 45.5 ± 0.1, 48.2 ± 0.3, and 54.3 ± 1.0 °C, respectively. The initial first phase *T*_m_ value for GTPγS was 45.5 ± 0.3. All data are expressed as mean ± SD.

Next, we selected RAS as a target protein to further study the one-step peptide-probe thermal protocol. We first mimicked the PLI-type stabilization of KRAS (500 nM) in both the absence and presence of MgCl_2_ (1 mM) (Figure 3A). Simultaneously, we studied the stability of the background signal, corresponding to the interaction of the Eu-probe with Q14 in the absence of the protein. At the selected concentrations, the background signal did not show any significant temperature-related changes. The observed Δ*T*_m_ in the absence and presence of MgCl_2_ (1 mM) was 9.1 °C, which is similar to the Δ*T*_m_ observed in the protein-probe assay (10.9 °C).^26^

We^26^ and others^34,35^ previously demonstrated that protein concentration can significantly impact not only protein detectability but also the observed *T*_m_ values. Thus, we determined *T*_m_ values of HRAS and NRAS at concentrations of 100, 250, and 500 nM in the one-step peptide-probe protocol; *T*_m_ values were slightly different and protein concentration-dependent (Figure S30). However, protein concentration dependency was not significant as we previously observed with the protein-probe.^26^ The *T*_m_ values were 46.4 ± 1.2–50.3 ± 6 °C, which matched that of KRAS (47.6 ± 0.6 °C) in the absence of Mg^2+^ (Figures S30 and 3A). However, the detectability of these RAS proteins varied with the protein concentration, with NRAS being more visible at lower concentrations compared to HRAS and especially KRAS. This finding contrasts with our original protein-probe assay, where KRAS showed higher detectability compared to other RAS proteins.^26^

To gain insight into the difference in detectability among the RAS proteins, even with the same Eu-probe, we conducted assays using two shorter versions of KRAS that lacked the high variable region (HVR).^26^ These truncated KRAS proteins showed higher detectability than full-length KRAS, and their *T*_m_ values (iMet-KRAS (2–169): 48.4 ± 1.5 °C; Ac-KRAS (2–169): 46.6 ± 1.0 °C) were consistent with expectations (Figure S31). These results indicate that the high KRAS detectability with the original protein-probe prefers the positively charged HVR on full-length KRAS, not present in HRAS and NRAS. However, the HVR of KRAS appears to have a negative effect on the current system, as full-length KRAS was less detectable than truncated KRAS, as well as full-length HRAS and NRAS.

To assess the compatibility of the one-step assay with the presence of various chemicals, we determined the *T*_m_ values of MDH (100 nM) in both the absence and presence of MgCl_2_ (2.5 mM), CaCl_2_ (1 mM), dithiothreitol (DTT, 10 mM), and glycerol (2.5%) using the one-step peptide-probe assays (Figure S32). The chemicals slightly increased the *T*_m_ value of MDH (1–2.2 °C) as expected because these chemicals have been reported to increase protein *T*_m_ values.^36−39^ The experiments were run under a typical concentration used in assay solutions, demonstrating the compatibility of the one-step peptide-probe for assaying protein stability.

We conducted further research on the thermal stabilization of nucleotide-binding proteins using guanosine nucleotides, with a focus on the α-subunit of the heterotrimeric GTPase GαS (R201C, C237S).^40^ The R201C mutation of GαS has been shown in multiple cancer types.^41^ To study thermal profiles, we incubated 100 nM GDP-loaded GαS (R201C, C237S) with 5 μM nucleotides, including GDP, GppNHp, and GTPγS, in a solution containing 10 mM HEPES at pH 7.5, 10 mM NaCl, 1 mM MgCl_2_, and 0.01% Triton X-100 (Figure 3B). The background-subtracted data revealed a *T*_m_ value of 44.4 °C for GαS (R201C and C237S) without any added nucleotide. The addition of GDP had a minimal effect on the thermal stability of GαS (R201C, C237S) (Δ*T*_m_ = 2 °C), while the nonhydrolyzable GTP analogs, GppNHp and GTPγS, led to a significant increase in thermal stability for GαS (R201C, C237S), with Δ*T*_m_ values of 3.9 and 10.0 °C, respectively. While the thermal profiles of GDP and GppNHp followed the single-phasic profile of the protein alone, GTPγS exhibited a two-phasic profile. Our interpretation is that, at higher temperatures, GαS (R201C, C237S) was only partially loaded with GTPγS, as the first phase followed the profile of the protein alone, with a nearly equal *T*_m_ value of 45.5 °C. The data clearly indicates that nucleotide exchange can be detected with the developed probes.

Current methods for thermal profiling of proteins require a high concentration, often in the micromolar range, and concentration-dependent changes in melting temperature are not always obvious. To address this, we conducted further studies on the concentration dependence of the tumor suppressor protein p53—core domain mutant M133L/V203A/N239Y/N268D (residues 94–312). We studied three proteins WT, R273C, and Y220C. The somatic p53 mutation Y220C is known to lower the thermal stability of the DNA-binding domain resulting in loss of occupancy to target gene promoters.^42^ Another p53 cysteine mutation, R273C, disrupts direct interactions with the DNA-phosphate backbone, providing lower affinity and promoter occupancy than the wild type. *T*_m_ values were measured for p53_wt_, p53_R273C_, and p53_Y220C_ at significantly lower concentrations (125, 250, 500, and 1000 nM) than those used in current methods (Figure S33).^43^ At high protein concentration, the profiles are clearly one-phasic, while at lower concentrations, the profiles exhibit two phases, indicating fine-structural melting profiling of the dimeric proteins (more details in the caption of Figure S33 and within Table S2). Concerning the first melting phase, we observed a clear concentration-dependent shift in *T*_m_ values (125 vs 1000 nM), with a Δ*T*_m_ of 2.9, 3.5, and 3.7 °C for p53_wt_, p53_R273C_, and p53_Y220C_, respectively. The S/B ratio remained consistently above 5 at all concentrations, indicating reliable assays. The *T*_m_ values of the three proteins were also measured using the DSF method and compared to the peptide-probe data as presented in Table S2. Generally, lower *T*_m_ values were measured with the DSF method compared to the developed method.

**Figure 4:**
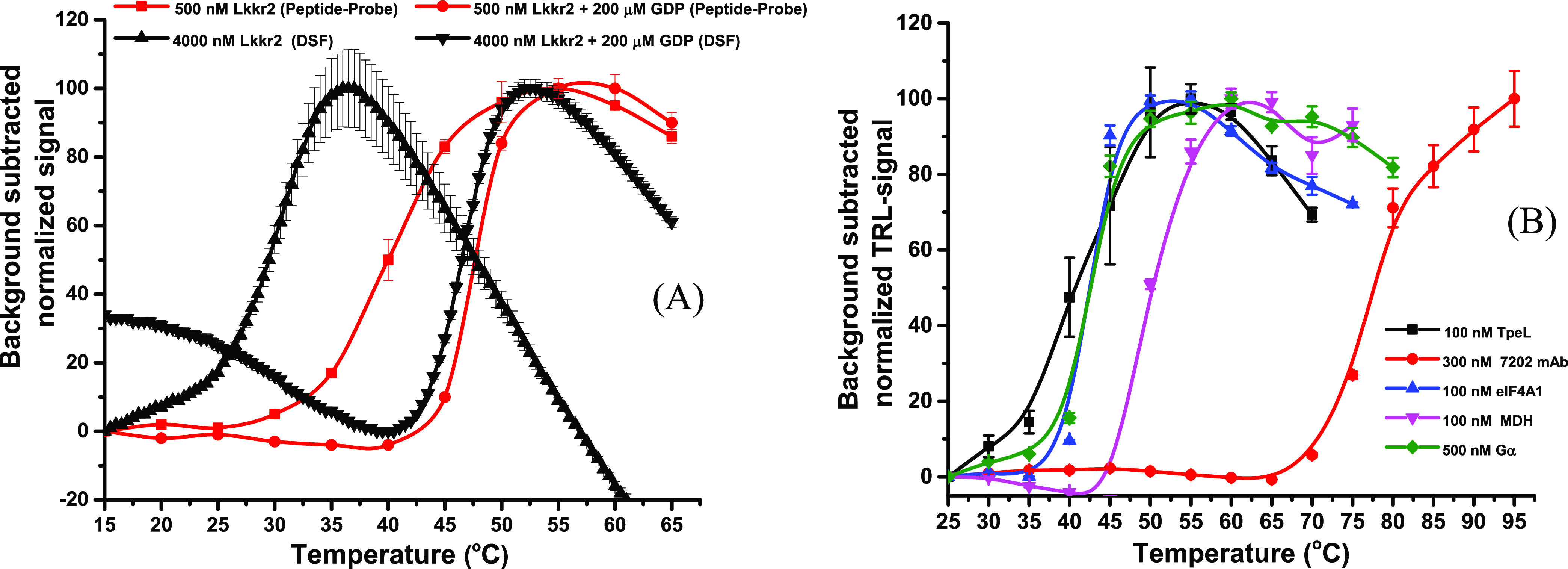
(A) Melting curves of Lrrk2 (wt) were measured in both the absence and presence of 200 μM GDP using the one-step peptide-probe and DSF methods. The one-step peptide-probe assay yielded *T*_m_ values of 40.2 ± 0.1 and 47.7 ± 0.1 for Lrrk2 (500 nM) without and with GDP, respectively. In comparison, DSF resulted in *T*_m_ values of 30.4 ± 0.1 and 46.5 ± 0.0 for Lrrk2 (4000 nM) without and with GDP, respectively. (B) Melting curves of various proteins, including TpeL, 7202 mAb, elF4A1, MDH, and G(i)α, were measured using the one-step peptide-probe assay with the Eu-probe (1 nM) and Q14 (10 nM) in HEPES buffer (10 mM, pH 7.5, 10 mM NaCl, 0.01% Brij 30). The *T*_m_ values for TpeL, 7202 mAb, elF4A1, MDH, and G(i)α were 46.9 ± 0.7, 77.3 ± 0.4, 42.4 ± 0.2, 50.0 ± 0.2, and 42.9 ± 0.3 °C, respectively. The data are presented as the mean ± SD.

We also compared the temperature profiles of the Lrrk2 GTPase domain using the one-step peptide-probe and DSF methods with and without 200 μM GDP. The DSF data indicated a *T*_m_ value of 30.3 ± 0.1 °C (S/B: 1.4) and 46.5 ± 0.0 °C (S/B: 1.7), with GDP, while the one-step peptide-probe method resulted in a *T*_m_ value of 40.2 ± 0.1 °C (S/B: 4.4) and 47.7 ± 0.1 °C (S/B: 3.6) (Figure 4A). The higher *T*_m_ value for Lrrk2 without GDP using the one-step peptide-probe method can be attributed, in part, to the lower protein concentration (500 nM) compared to DSF (4000 nM). GDP significantly increases the protein stability and lowers the assay variation. The higher signal variation without GDP can be explained by the low stability of the protein. These results demonstrate the versatility of the one-step peptide-probe method as a label-free, time-resolved luminescence technique for determining *T*_m_ values of a variety of proteins.

Finally, we investigated the universality of the method by determining *T*_m_ values for five additional proteins: TpeL (100 nM), 7202 mAb (300 nM), eIF4A1 (100 nM), MDH (100 nM), and G(i)α (500 nM). These proteins were selected to cover a wide range of molecular weights and *T*_m_ values. The *T*_m_ values measured for these proteins were 46.9 ± 0.7, 77.3 ± 0.4, 42.4 ± 0.2, 50.0 ± 0.2, and 42.9 ± 0.3 °C, respectively (Figure 4B).

## Conclusions

In this study, we synthesized and evaluated multiple quencher structures to measure protein thermal profiles at neutral pH using a nanomolar protein concentration in the one-step peptide-probe assay. We modified the parent Cy5 quencher structure to improve the assay performance, providing assay conditions suitable for protein thermal studies without the risk of pH-induced protein denaturation. The new protocol is faster and more economical and enables one-step assays, making it suitable for high-throughput screening applications compared to our previous two-step method.

We found that steric hindrance and electrostatic interactions are more important than hydrophobic interactions in the interaction between the Eu-probe and the quenchers. Increasing the number of positive charges in the quencher structure improved the Eu-probe quenching properties, likely due to the luminescent Eu-probe having a negative net charge opposite to that of the quencher. These findings may be useful in the development of new donors and acceptors for fluorescence resonance energy transfer (FRET).

Our novel one-step peptide-probe assay scheme provided surprising information at low protein concentrations as one-phasic melting curves gradually changed to two-phasic curves for dimeric p53 proteins. This suggests that new methodologies with sensitivity higher than that of existing technologies are required to obtain additional fine-structural data.

In this study, we focused on modifying the quencher structure, but further improvements in the assay can be made by changing the peptide structure of the Eu-probe in future work.

## Supporting Information Available

Detailed experimental procedures, materials and methods, HPLC traces of the quenchers, and excitation and emission spectra of the probes, along with melting temperature curves of proteins (PDF)
