# Supplementary material for: Nanomolar Protein Thermal Profiling with Modified Cyanine Dyes
Source: Anal Chem. 2023 Dec 7;95(50):18344–51. doi: 10.1021/acs.analchem.3c02844 (PMC10733900; doi:10.1021/acs.analchem.3c02844)
Supplement: Supplementary file 1 — ac3c02844_si_001.pdf [file ac3c02844_si_001.pdf]

SUPPORTING INFORMATION

Nanomolar Protein Thermal Profiling with Modified Cyanine Dyes

Morteza Malakoutikhah,[a*] Randa Mahran,[a] Negin Gooran,[a] Ahmadreza Masoumi,[a] Katri Lundell,[b] Arto Liljeblad,[b] Keelan Guiley,[c, d] Shizhong Dai,[c, e] Qinheng Zheng,[c] Lawrence Zhu,[c] Kevan M. Shokat,[c] Kari Kopra,[a] and Harri Härmä[a, c]

[a] Department of Chemistry, University of Turku, Henrikinkatu 2, 20500 Turku, Finland

[b] Laboratory of Synthetic Drug Chemistry, Institute of Biomedicine, University of Turku, Kiinamyllynkatu 10, FI-20520 Turku, Finland

[c] Department of Cellular and Molecular Pharmacology and Howard Hughes Medical Institute, University of California, San Francisco, California 94158, USA

[d] Current address: Rezo Therapeutics, Inc., San Francisco, California 94158, USA

[e] Current address: Department of Genetics, Stanford University, Stanford, CA, USA

E-mails: randa.r.mahran@utu.fi (R.M.), negin.gooran@utu.fi (N.G.) ahmadreza.masoumihossein@utu.fi (A.M.), katlun@utu.fi (K.L.), artlilje@utu.fi (A.L.), keelanguiley@gmail.com (K.G.), szdai@stanford.edu (S.D.), Qinheng.Zheng@ucsf.edu (Q.Z.), Lawrence.Zhu@ucsf.edu (L.Z.), kevan.shokat@ucsf.edu (K.S.), khkopr@utu.fi (K.K.), harri.harma@utu.fi (H.H.),

*Corresponding author email: morteza.malakoutikhah@utu.fi

Contents:

**Materials and methods**	S-3
**Materials and instrumentation**	S-3
**One-step Peptide-Probe using 96-well plates**	S-4
**DSF Assay**	S-4
**Eu-probe preparation**	S-4
**Quenchers Preparation**	S-5
**RP-HPLC Purification**	S-7
**Data analysis**	S-7
**Figure S1**. Two-step Protein-Probe and one-step Peptide-Probe protocols	S-8
**Figure S2**. HPLC trace (monitored at 254 nm) of Q2	S-8
**Figure S3**. HPLC trace (monitored at 254 nm) of Q3	S-8

Figure S4. Comparison of quenching efficiency of Q1-Q7 with the Eu-probe S-9
Figure S5. Comparison of S/B ratios in Protein-Probe of Q1-Q10 with the Eu-probe S-9
Figure S6. HPLC trace (monitored at 254 nm) of Q8 S-9
Figure S7. HPLC trace (monitored at 254 nm) of Q9 S-10
Figure S8. HPLC trace (monitored at 254 nm) of Q10 S-10
Figure S9. HPLC trace (monitored at 254 nm) of Q11 S-10
Figure S10. HPLC trace (monitored at 254 nm) of Q12 S-10
Figure S11. HPLC trace (monitored at 254 nm) of Q13 S-11
Figure S12. HPLC trace (monitored at 254 nm) of Q14 S-11
Figure S13. Comparison of S/B ratios in Protein-Probe of Q1, Q7, Q11-Q14 with the Eu-probe S-11
Figure S14. TRL-signals of Eu-probe in the absence and presence of Q1, Q7, Q13 S-12
Figure S15. TRL-signals of Eu-chelate in the absence and presence of Q1, Q7, Q13 S-12
Figure S16. S/B ratios of Protein-Probe antibody denaturation assays using Eu-probe and Q13 S-12
Figure S17. TRL signals of Eu-probe in the presence of the Q1 and Q14 S-13
Figure S18. S/B ratios of Protein-Probe antibody denaturation assays using Eu-probe and Q14 S-13
Figure S19. Normalized excitation spectra of Q1-Q7 in DMSO S-13
Figure S20. Normalized excitation spectra of Q1-Q7 in H_2O S-14
Figure S21. Normalized emission spectra of Q1-Q7 in DMSO S-14
Figure S22. Normalized emission spectra of Q1-Q7 in H_2O S-14
Figure S23. Normalized excitation spectra of Q1, Q8-Q10 in DMSO S-14
Figure S24. Normalized emission spectra of Q1, Q8-Q10 in DMSO S-15
Figure S25. Normalized excitation spectra of Q1, Q11-Q14 in DMSO S-15
Figure S26. Normalized emission spectra of Q1, Q11-Q14 in DMSO S-15
Figure S27. Normalized excitation spectra of Q14 in the both absence and presence of Eu-probe and MDH S-15
Figure S28. Normalized emission spectra of Q14 in the both absence and presence of Eu-probe and MDH S-16
Figure S29. Melting curves of BCA in the both absence and presence of its inhibitor acetazolamide S-16
Table S1. Melting temperature (T_m) values of bovine carbonic anhydrase (BCA II) S-16
Figure S30. Melting curves of HRAS and NRAS at different concentrations S-17
Figure S31. Melting curves of Ac-KRAS (2-169) and iMet-KRAS (2-169) S-17
Figure S32. Melting curves of MDH in the both absence and presence of $MgCl_2$, $CaCl_2$, DTT and glycerol S-17
Figure S33. Melting curves of $p53_{wt}$, $p53_{R273C}$ and $p53_{Y220C}$ at different concentrations S-18
Table S2. Comparison of Thermal Profile Data for $p53_{wt}$, $p53_{R273C}$, and $p53_{Y220C}$ S-19
References S-19

Materials and methods

Materials and instrumentation

The nonadentate Eu(III)-chelate, {2,2',2'',2'''-{[4'-(4'''-isothiocyanatophenyl)-2,2',6',2''-terpyridine-6,6''-diyl]bis(methylene nitrilo)}tetrakis(acetate)}europium(III) was from QRET Technologies (Turku, Finland) and conjugated according to manufacturer's instruction to the probe peptide. Probe peptide (H_2N-EYEEEEEVEEEVEEE) was purchased from Pepmic Co., Ltd (Suzhou, China). 1,1,3,3,3′,3′-hexamethylindodicarbocyanine iodide (Q1), ethanolamine, hexylamine, ethylenediamine, analytical-grade solvents and buffer components were purchased from Sigma-Aldrich (St. Louis, MO). Cyanine5 NHS ester, sulfo-cyanine5 carboxylic acid (Q4), cyanine5.5 carboxylic acid (Q5), cyanine5 carboxylic acid (Q6), cyanine5 carboxylic amine (Q7) were supplied by Lumiprobe GmbH (Germany, Hannover).

Peptides Ac-KGGGRGGGR-$CONH_2$, Ac-KGGGRGGGRRGGGRGGGR-$CONH_2$, Ac-KRRRRRR-$CONH_2$ and Ac-KRRRRRRRRRRRR-$CONH_2$ were supplied by Synpeptide Co., Ltd (Shanghai, China). Target proteins; antibodies, and carbonic anhydrase from bovine erythrocytes (BCA II) were purchased from Medix Biochemica (Espoo, Finland), BioSpa (Milan, Italy), Sigma-Aldrich (St. Louis, MO, USA), Roche (Basel, Switzerland), and Merck (Darmstadt, Germany), respectively. *E. coli* expression, and purification of TpeL, iMet-KRAS (1-169), Ac-KRAS (1-169), KRAS (1-166), KRAS (1-169), KRAS (2-188), NRAS (1-189), and HRAS (1-189) is described elsewhere and proteins were a kind gift from Leidos Biomedical Research, Inc., Frederick National Laboratory for Cancer Research .[1-3] Eukaryotic initiation factor 4A (eIF4A) was produced and purified at the Medical Research Council (MRC) Toxicology Unit, using their constructs and protocols with minor changes.[4,5] N-terminally HIS-tagged human G(i)α (Uniprot #P63096) was produced in *E. coli,* and it was a kind gift from Arto Pulliainen (University of Turku).[6] Triethylammonium acetate (TEAA) was from PanReac Applichem (Darmstadt, Germany). Acetazolamide (AZA) was from Alfa Aesar, Thermo Fisher Scientific (Haverhill, MA, United States). All assays without heating were performed at black OptiPlate 384-well from PerkinElmer (384-F). Thermal assays were performed either at 96- or 384-well microtiter plates from 4titude (Surrey, U.K.). $EuCl_3$ standard, and DELFIA enhancement solution (DES) were purchased from PerkinElmer Life and Analytical Sciences, Wallac (Turku, Finland).

Time-resolved luminescence (TRL) measurements were performed with Tecan Spark 20M (Tecan Life Sciences, Männedorf, Switzerland), using 340 nm excitation wavelength, 620 nm emission wavelength, 400 µs integration time and 800 µs delay time. A PTC-100

Programmable Thermal Controller (MJ Research, Inc., Watertown, MA) was used for thermal ramping.

One-step Peptide-Probe using 96-well plates

Melting curves of proteins were monitored in one-step assays where 8 μL of a protein (300 nM in 0.1x PBS pH 7.5) or protein + Acetazolamide (300 nM + 5 μM in 0.1x PBS pH 7.5) sample was added to a following with 65 μL of the modulation solution in Tris (10 mM, pH 7.5, 0.01% Triton X-100) containing the Eu-probe (1 nM) and the quencher **14** (12.5 nM). Then the mixture was heated at the desired temperatures (ranging 25-85° C with a temperature step interval 2-5° C) and TRL-signal is immediately measured after each temperature.

DSF Assay

Melting curves of Lrrk2 (4 μM) were monitored in a DSF assay both in the absence and presence of GDP (200 μM). In this assay, 25 μL of Lrrk2 (4 μM) or Lrrk2 + GDP (4 μM + 200 μM) in a buffer consisting of 50 mM HEPES pH 7.4, 62.5 mM NaCl, 5 mM $MgCl_2$, 5% glycerol, and 4X SYPRO Orange dye were added to a 96-well plate. Subsequently, the mixtures were heated at the desired temperatures, ranging from 15°C to 65°C with a temperature step interval of 1°C, and luminescence signals were immediately measured after each temperature increment by the BioRad C1000 thermocycler unit coupled to a CFX96 Optical Reaction Module.

Melting curves of $p53_{wt}$, $p53_{R273C}$, and $p53_{Y220C}$, each at a concentration of 2 μM, were monitored in a DSF assay. In this assay, 25 μL of $p53_{wt}$, $p53_{R273C}$, and $p53_{Y220C}$ in a buffer containing 50 mM HEPES pH 7, 150 mM NaCl, 1 mM DTT, and 2X SYPRO Orange dye were added to a 96-well plate. Subsequently, the mixtures were heated at the desired temperatures, ranging from 15°C to 65°C with a temperature step interval of 1°C, and luminescence signals were immediately measured after each temperature increment by the BioRad C1000 thermocycler unit coupled to a CFX96 Optical Reaction Module

Eu-probe preparation

Isothiocyanate activated Eu(III)-chelate was conjugated to the N-terminus of the peptide sequence to prepare the Eu-probe. Eu(III)-chelate conjugations was performed in labeling buffer containing pyridine/H_2O/triethylamine in 9:1.5:0.1 ratio. Eu(III)-chelate (1 mg) was dissolved in 100 μL of water and mixed with the probe peptide (0.5 mg) in 100 μL of the labeling buffer. Reaction solution was incubated at room temperature (RT) for 18 h. Eu-probe purification was carried out using reversed-phase adsorption chromatography, Dionex ultimate 3000 LC system from Thermo Fischer Scientific, Dionex, and Ascentis RP-amide C18 column from Sigma-Aldrich, Supelco Analytical under the following conditions: eluent system

containing 50 mM TEAA pH 7.0:ACN 100%, linear gradient (1 ml/min from 10:90 to 50:50 in 17 min). After purification, Eu-probe concentration was determined based on the Eu(III)-ion concentration by comparing observed TRL-signal to a commercial Eu(III)-standard (DELFIA), assuming that the probe peptide and Eu(III)-chelate are in 1:1 ratio.

Quenchers Preparation

The quenchers **2-3** were synthesized according to the procedures described by others, in a two-step reaction.[7-10]

Quencher 2. First step: 2,3,3-Trimethylindolenine (160 µL, 1 mmol) and iodobutane (136 µL mL, 1.2 mmol) were added to a microwave reaction vial equipped with a micro magnetic stir bar. The microwave vial then was sealed with a camp and heated in microwave oven at 155 °C for 30 min. After cooling, the resulting residue was washed with ether/aceton, however, unlike all the procedures we could not isolate the desired product as a solid but an oily sticky material, which was used as a starting material in the second step of the reaction.

Second step: a mixture of the crude product of the first step (68.6, 0.2 mmol), bis-iminium salt (34.3 mg, 0.13 mmol), NaOAc (28.9 mg, 0.4 mmol) and acetic anhydride (1.7 mL) was placed in sealed microwave vessel with stirring bar. Sealed vessel was heated in in microwave oven at 150 °C for 20 min. Reaction mixture was diluted with diethyl ether (10-20 mL) and filtered in vacuo. Solid was washed twice with diethyl ether (5 mL). A clean filter flask was attached to the funnel and the resulting solid was dissolved with dichloromethane (10-15 mL) leaving unreacted sodium acetate crystals on the filter funnel. The filtrate was transferred to a clean round bottom flask and dichloromethane was removed with a rotary evaporator. The blue/green crude was formed after solvent removal. The crude product was purified by reverse-phase HPLC. MS calculated for $C_{33}H_{43}N_2^+$ 467.34 found 467.32.

Quencher 3. First step: 2,3,3-Trimethylindolenine (32 µL, 0.2 mmol) and benzyl bromide (47.5 µL mL, 0.4 mmol) were added to a microwave reaction vial equipped with a micro magnetic stir bar. The microwave vial then was sealed with a camp and heated in microwave oven at 130 °C for 30 min. After cooling, the resulting residue was washed with ether/aceton, however, unlike all the procedures we could not isolate the desired product as a solid but an oily sticky material, which was used as a starting material in the second step of the reaction.

Second step: a mixture of the crude product of the first step (65.8, 0.2 mmol), bis-iminium salt (25.9 mg, 0.1 mmol), NaOAc (22 mg, 0.3 mmol) and acetic anhydride (1.3 mL) was placed in sealed microwave vessel with stirring bar. Sealed vessel was heated in in microwave oven at 150 °C for 20 min. Reaction mixture was diluted with diethyl ether (10-20 mL) and filtered in vacuo. Solid was washed twice with diethyl ether (5 mL). A clean filter flask was attached to

the funnel and the resulting solid was dissolved with dichloromethane (10-15 mL) leaving unreacted sodium acetate crystals on the filter funnel. The filtrate was transferred to a clean round bottom flask and dichloromethane was removed with a rotary evaporator. The blue crude was formed after solvent removal. The crude product was purified by reverse-phase HPLC. MS calculated for $C_{39}H_{39}N_2^+$ 535.31 found 535.31.

The quenchers **8-14** were prepared using Cyanine5 NHS ester (3H-Indolium, 2-[5-(1,3-dihydro-1,3,3-trimethyl-2H-indol-2-ylidene)-1,3-pentadien-1-yl]-1-[6-[(2,5-dioxo-1-pyrrolidinyl)oxy]-6-oxohexyl]-3,3-dimethyl-, tetrafluoroborate) as a labelling agent.

Quencher 8. Cyanine5 NHS ester (0.25 mg, 0.37 μmol) in 25 μL DMSO was added to ethanolamine (0.113 μL, 1.87 μmol) in DMSO (25 μL) and disodium phosphate buffer (50 μL, 50 mM, pH 8) in a 1.5 mL tube. The reaction tube was left on shaker at room temperature for 2 h. The resulting product was purified with by reverse-phase HPLC. MS calculated for $C_{34}H_{44}N_3O_2^+$ 526.34 found 526.3.

*Quencher 9***.** Cyanine5 NHS ester (0.25 mg, 0.37 μmol) in 25 μL DMSO was added to ethylenediamine (0.124 μL, 1.87 μmol) in DMSO (25 μL) and disodium phosphate buffer (50 μL, 50 mM, pH 8) in a 1.5 mL tube. The reaction tube was left on shaker at room temperature for 2 h. The resulting product was purified with by reverse-phase HPLC. MS calculated for $C_{34}H_{45}N_4O^+$ 525.36 found 525.4.

Quencher 10. Cyanine5 NHS ester (0.25 mg, 0.37 μmol) in 25 μL DMSO was added to hexylamine (0.245 μL, 1.87 μmol) in DMSO (25 μL) and disodium phosphate buffer (50 μL, 50 mM, pH 8) in a 1.5 mL tube. The tube was left on shaker at room temperature for 2 h. The resulting product was purified with by reverse-phase HPLC. MS calculated for $C_{38}H_{52}N_3O^+$ 566.41 found 566.4.

Quencher 11. Cyanine5 NHS ester (0.25 mg, 0.37 μmol) in 10 μL DMSO was added to peptide Ac-KRRRRRR-CONH$_2$ (0.41 mg, 0.37 μmol) in DMSO:H_2O (20 μL) and disodium phosphate buffer (70 μL, 100 mM, pH 8) in a 1.5 mL tube. The tube was left on shaker at room temperature overnight. The resulting product was purified with by reverse-phase HPLC. MS calculated for $C_{76}H_{126}N_{29}O_9^+$ 1589.03 found 795 $[M+H]^{2+}$, 530.5 $[M+2H]^{3+}$, 398.2 $[M+3H]^{4+}$, 318.8 $[M+4H]^{5+}$, 265.9 $[M+5H]^{6+}$.

Quencher 12. Cyanine5 NHS ester (0.25 mg, 0.37 μmol) in 10 μL DMSO was added to peptide Ac-KRRRRRRRRRRRR-CONH$_2$ (0.75 mg, 0.37 μmol) in DMSO:H_2O (20 μL) and disodium phosphate buffer (70 μL, 100 mM, pH 8) in a 1.5 mL tube. The reaction tube was left on shaker at room temperature overnight. The resulting product was purified with by reverse-phase

HPLC. MS calculated for $C_{112}H_{198}N_{53}O_{15}^+$ 2527.17 found 842.8 $[M+2H]^{3+}$, 632.5 $[M+3H]^{4+}$, 506.2 $[M+4H]^{5+}$, 422.0 $[M+5H]^{6+}$, 361.9 $[M+6H]^{7+}$, 316.7 $[M+7H]^{8+}$, 281.8 $[M+8H]^{9+}$.

Quencher 13. Cyanine5 NHS ester (0.25 mg, 0.37 μmol) in 10 μL DMSO was added to peptide Ac-KGGGRGGGR-$CONH_2$ (0.31 mg, 0.37 μmol) in DMSO:H_2O (15 μL) and disodium phosphate buffer (75 μL, 100 mM, pH 8) in a 1.5 mL tube. The reaction tube was left on shaker at room temperature overnight. The resulting product was purified with by reverse-phase HPLC. MS calculated for $C_{64}H_{96}N_{19}O_{11}^+$ 1306.75 found 654.0 $[M+H]^{2+}$, 436.4 $[M+2H]^{3+}$.

Quencher 14. Cyanine5 NHS ester (0.25 mg, 0.37 μmol) in 10 μL DMSO was added to peptide Ac-KGGGRGGGRRGGGRGGGR-$CONH_2$ (0.61 mg, 0.37 μmol) in DMSO:H_2O (30 μL) and disodium phosphate buffer (60 μL, 100 mM, pH 8) in an 1.5 mL tube. The reaction tube was left on shaker at room temperature overnight. The resulting product was purified with by reverse-phase HPLC. MS calculated for $C_{94}H_{150}N_{37}O_{20}^+$ 2118.47 found 706.8 $[M+2H]^{3+}$, 530.5 $[M+3H]^{4+}$, 424.5 $[M+4H]^{5+}$, 353.9 $[M+5H]^{6+}$.

RP-HPLC Purification. The crude quenchers were purified by reverse-phase HPLC using a C_4 column (150 mm × 4.6 mm × 5 μm, 200 Å, Phenomenex) with 1 mL/min flow with the following solvents: solvent A, 0.2 M TEAA in H_2O; solvent B, H_2O, solvent C, MeCN.

Data analysis

In all assays, the signal/background ratio (S/B) was calculated as μmax/μmin, where μmax is the signal of thermally denatured protein and μmin is the signal of native protein. The data were analyzed using Origin 2016 software (Origin Lab, Northampton, MA) with the standard sigmoidal fitting function:

y = A2 + (A1-A2)/(1 + (x/x0)^p).

For two-phase curves, T_m values were determined separately for each phase.

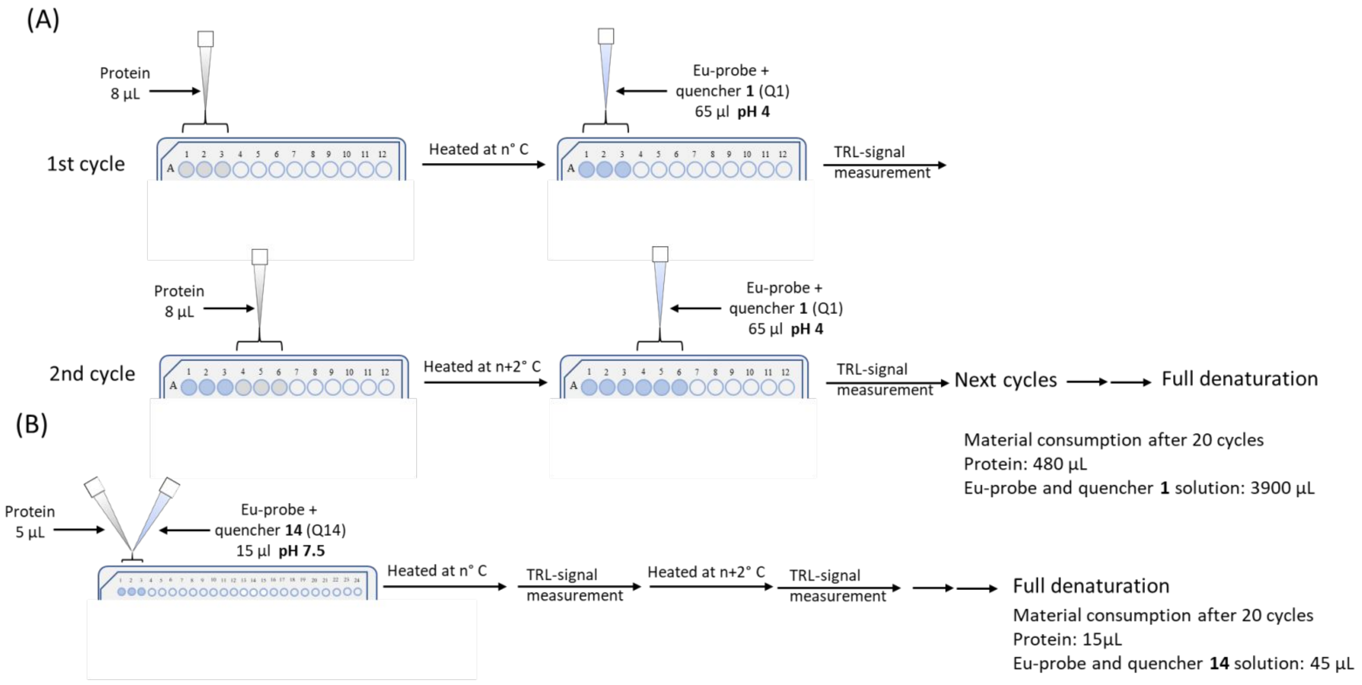

Figure S1. (A) Two-step Protein-Probe protocol: 1st cycle: Intact protein (8 µL) is heated at desired temperature, Eu-probe and quencher **1** (65 µl, pH 4) are added and TRL-signal is measured. 2nd cycle: addition of fresh intact protein (8 µL), Eu-probe and quencher (65 µl, pH 4) to new wells and heated followed by TRL measurement. Subsequent cycles: The same cycling continues until the protein is fully denatured. (B) One-step Peptide-Probe protocol: Intact protein (5 µL) and the Eu-probe and Q14 (15 µl, pH 7.5) are heated at desired temperature and measured for TRL-signal. The same wells are further heated at higher temperature and remeasured until protein is fully denatured.

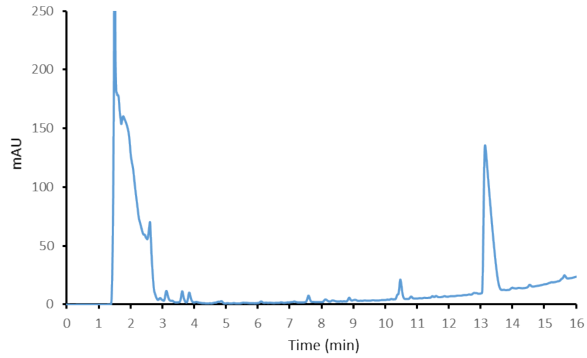

Figure S2. HPLC trace (monitored at 254 nm) of Q2. Gradient from 40% to 75% MeCN in 16 min. The retention time of the desired product was 13.3 min.

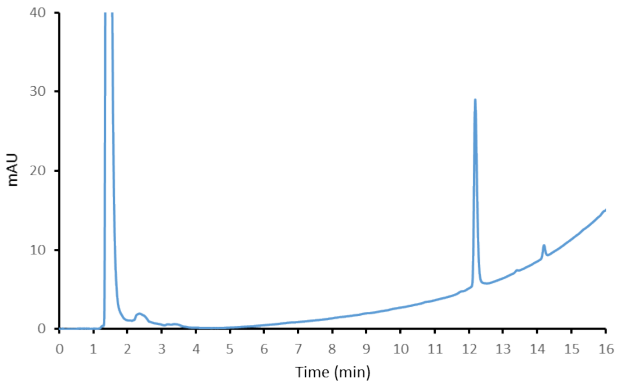

Figure S3. HPLC trace (monitored at 254 nm) of Q3. Gradient from 40% to 75% MeCN in 16 min. The retention time of the desired product was 12.2 min.

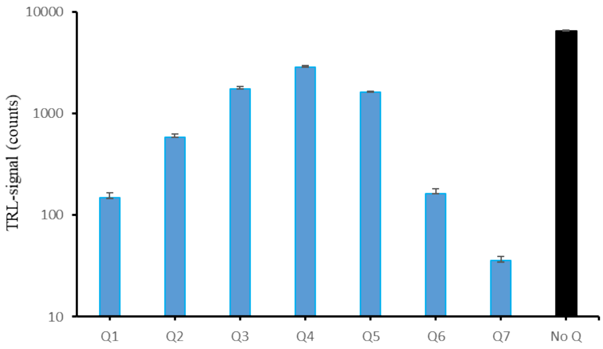

Figure S4. Comparison of quenching efficiency of Q1-Q7 with the Eu-probe: quenchers (1 µM) were mixed with the Eu-probe (1 nM) in citrate-phosphate buffer, pH 4, and their quenching ability was determined by measuring their TRL-signal. Significantly lower signal was observed with Q7 than Q1. This can be explained by the influence of either steric interferences of the larger side chain groups or high hydrophobicity of Q2-Q3. Beckford et al.[11] suggested that steric hindrance of side chain groups is a determining factor in binding between cyanine dyes and human serum albumin (HSA). In the case of Q7, the electrostatic interaction between positively charged side chain amine and the negatively charged peptide of the Eu-probe compensated for the side chain steric hindrance. In contrast, the presence of substituents in the indolenine rings of Q4-Q led to low quenching ability, which may be due to steric hindrance of substituents and/or electrostatic repulsion between negatively charged sulfate groups and the peptide of the Eu-probe. Obviously, the negative charge of Q6 also diminishes Eu-probe/quencher interaction. Black column represents Eu-probe TRL-signal without a quencher.

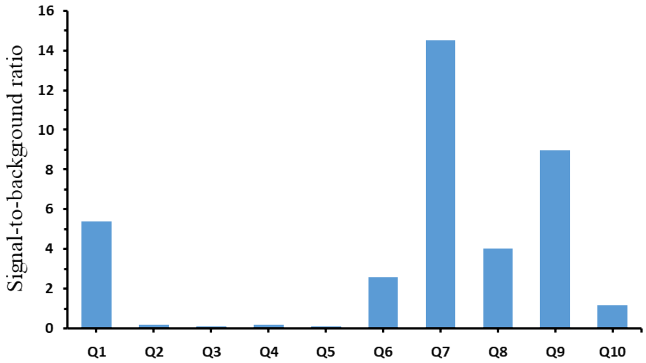

Figure S5. Comparison of S/B ratios in Protein-Probe antibody denaturation assays using the Eu-probe (1 nM) and quenchers Q1-Q10 (1 µM). Q7 exhibited the highest S/B ratio. Q8 showed a lower S/B ratio than Q9, indicating that the presence of a hydroxyl group on the indolenine side chain (Q8) was less effective. The hydroxyl group does not carry a positive charge at the assay pH of 4, unlike the amino group, leading to a weaker interaction with the Eu-probe. Furthermore, eliminating the amino group but retaining the C6 alkyl chain structure (Q10) of Q7 caused a significant decline in the S/B ratio compared to Q7. The S/B ratio was calculated by dividing the mean TRL-signal measured with the denatured sample at 85 °C by that of the intact sample in the antibody assay.

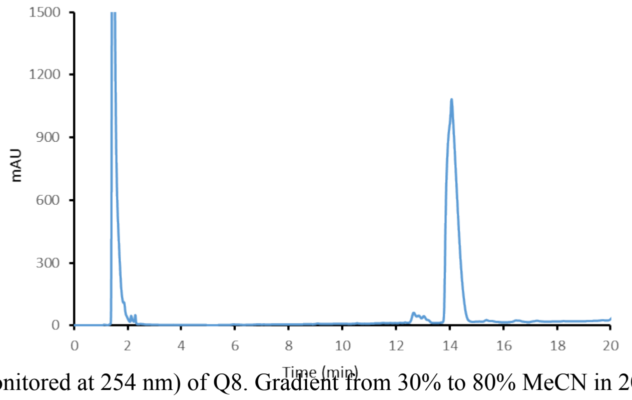

Figure S6. HPLC trace (monitored at 254 nm) of Q8. Gradient from 30% to 80% MeCN in 20 min. The retention time of the desired product was 14.2 min.

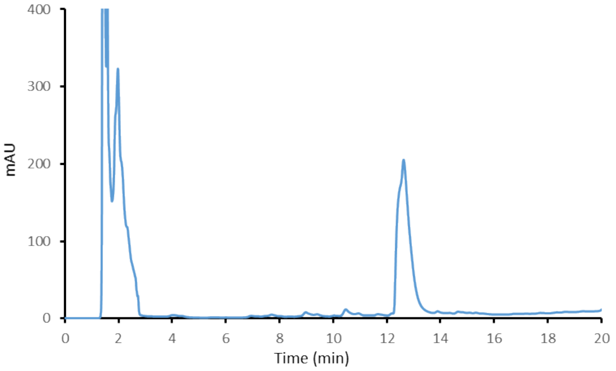

Figure S7. HPLC trace (monitored at 254 nm) of Q9. Gradient from 30% to 80% MeCN in 20 min. The retention time of the desired product was 12.6 min.

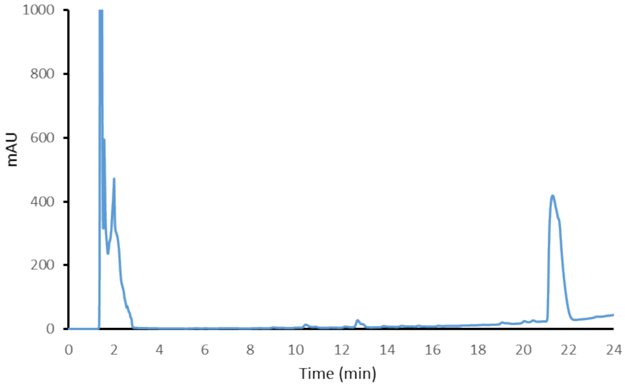

Figure S8. HPLC trace (monitored at 254 nm) of Q10. Gradient from 30% to 80% MeCN in 22 min. The retention time of the desired product was 21.3 min

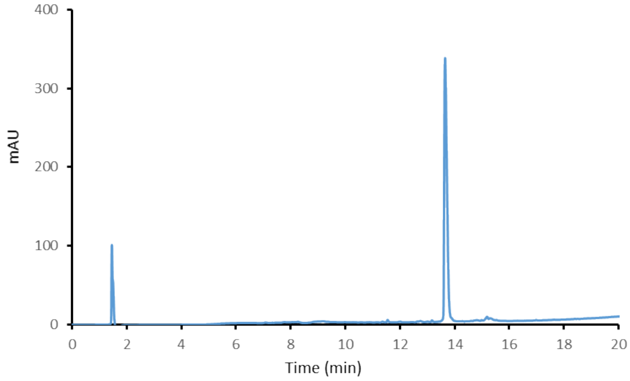

Figure S9. HPLC trace (monitored at 254 nm) of Q11. Gradient from 0% to 80% MeCN in 20 min. The retention time of the desired product was 13.7 min.

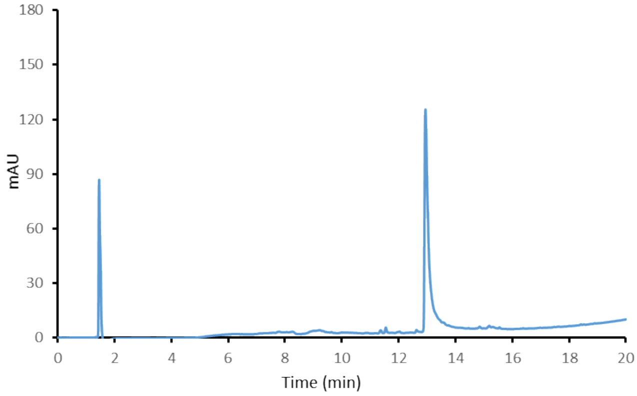

Figure S10. HPLC trace (monitored at 254 nm) of Q12. Gradient from 0% to 80% MeCN in 20 min. The retention time of the desired product was 13 min.

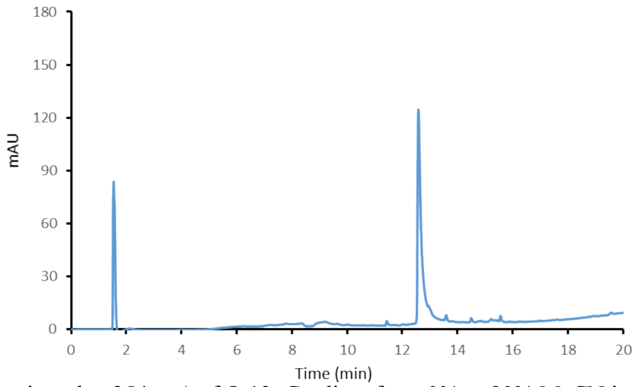

Figure S11. HPLC trace (monitored at 254 nm) of Q 13. Gradient from 0% to 80% MeCN in 20 min. The retention time of the desired product was 12.6 min.

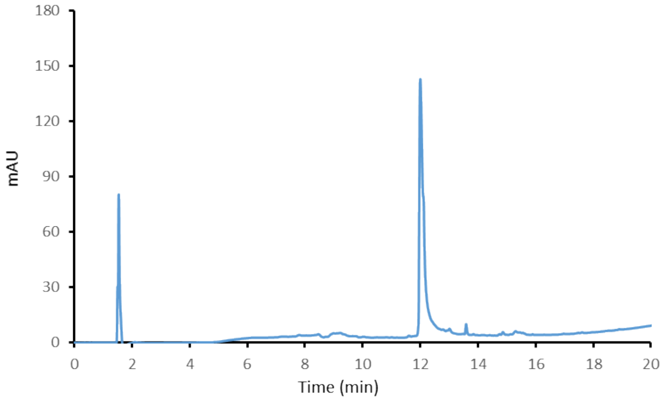

Figure S12. HPLC trace (monitored at 254 nm) of Q 14. Gradient from 0% to 80% MeCN in 20 min. The retention time of the desired product was 12 min.

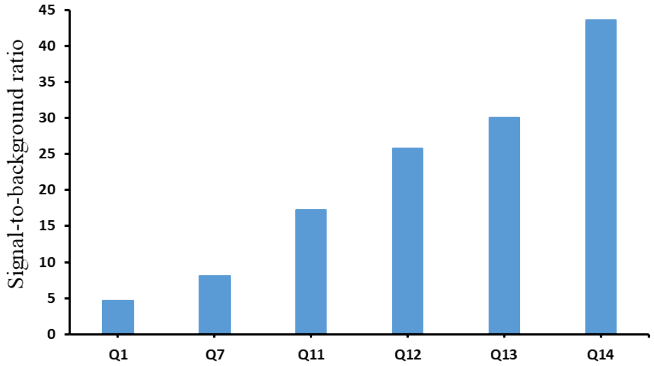

Figure S13. Comparison of S/B ratios in Protein-Probe antibody denaturation assays using the Eu-probe (1 nM) and quenchers Q1, Q7, Q11-Q14 (1 µM). The S/B ratio was calculated by dividing the mean TRL-signal measured with the denatured sample at 85 °C by that of the intact sample in the antibody assay.

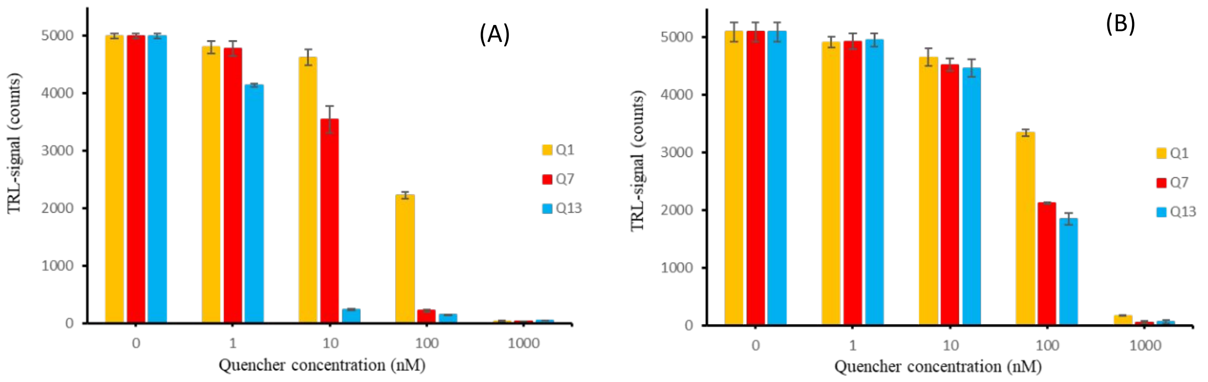

Figure S14. TRL-signals of the Eu-probe (1 nM) in the absence and presence of Q1, Q7, Q13 at four different concentrations (1, 10, 100, 1000 nM) at (A) H_2O (pH 7) and (B) a phosphate-citrate buffer (pH 3.4), both supplemented with 0.01% Triton X-100. Data are expressed as the mean ± SD.

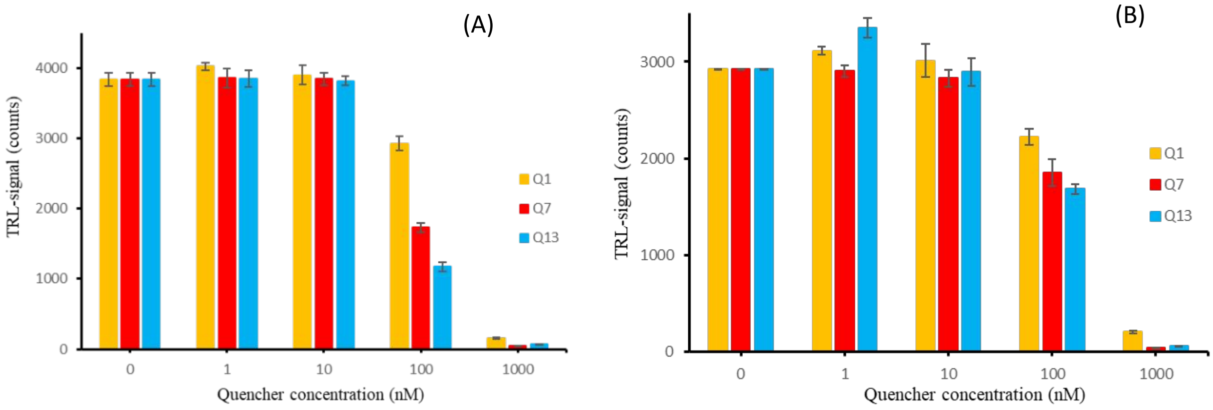

Figure S15. TRL-signals of the Eu-chelate (1 nM) in the absence and presence of Q1, Q7, Q13 at four different concentrations (1, 10, 100, 1000 nM) at (A) H_2O (pH 7) and (B) a phosphate-citrate buffer (pH 3.4), both supplemented with 0.01% Triton X-100Data are expressed as the mean ± SD.

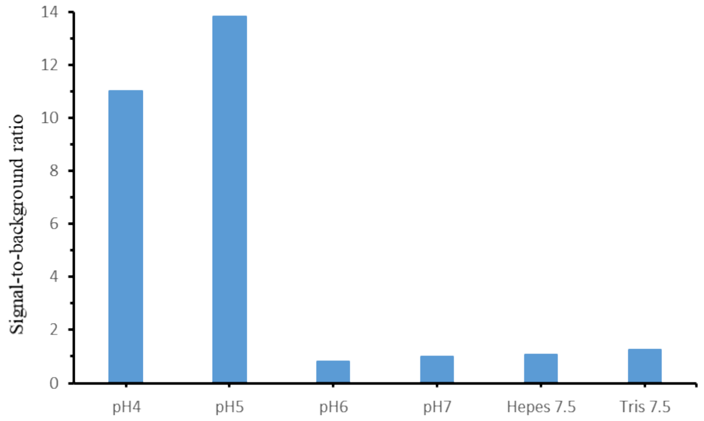

Figure S16. S/B ratios of Protein-Probe antibody denaturation assays using the Eu-probe (1 nM) and Q13 (1 µM) across various buffer conditions (phosphate-citrate pH 4-7; HEPES 10 mM pH 7.5; Tris 10 mM pH 7.5), all supplemented with 0.01% Triton X-100. The S/B ratio was calculated by dividing the mean TRL-signal from the denatured sample at 85 °C by that of the native antibody sample kept at room temperature.

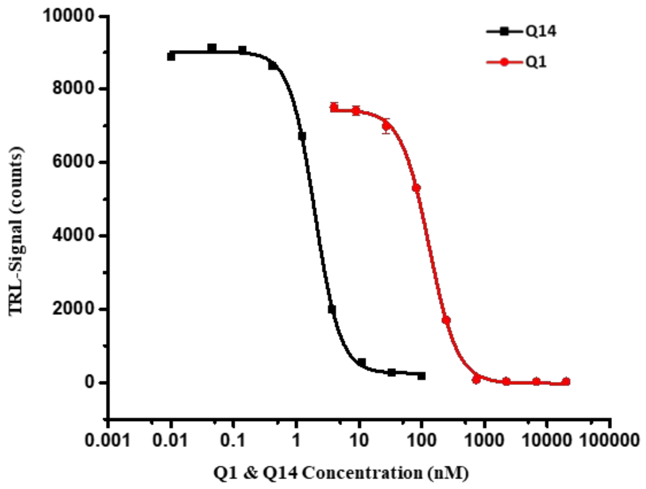

Figure S17. TRL signals of the Eu-probe (1 nM) in the presence of different concentrations of Q1 (red) and Q14 (black) at H_2O (pH 7), supplemented with 0.01% Triton X-100. The EC_{50} values of Q1 and Q14 were 131.0 ± 3.9 and 1.9 ± 0.05 nM, respectively.

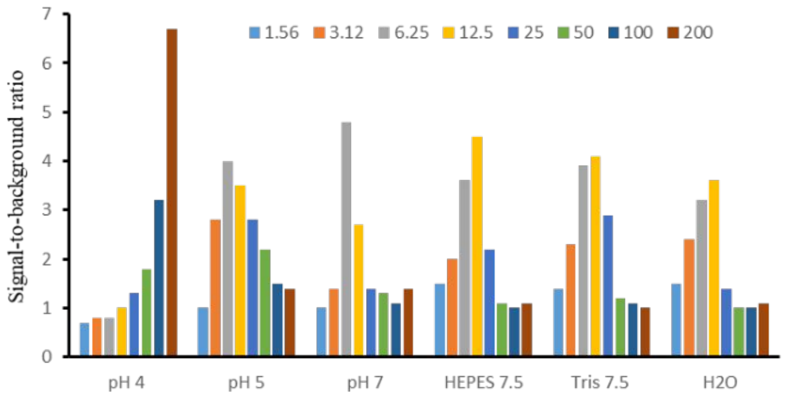

Figure S18. S/B ratios of Protein-Probe antibody denaturation assays using the Eu-probe and Q14 at different concentrations (1.56-200 nM) in various buffer conditions (phosphate-citrate pH 4, 5, 7; HEPES 10 mM pH 7.5; Tris 10 mM pH 7.5; H_2O) all supplemented with 0.01% Triton X-100. The S/B ratio was calculated by dividing the mean TRL-signal from the denatured sample at 85 °C by that of the native antibody sample kept at room temperature.

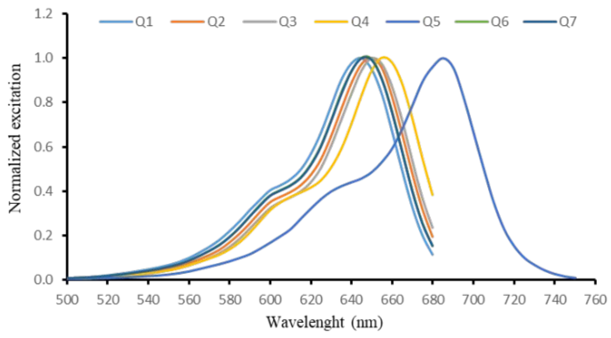

Figure S19. Normalized excitation spectra of Q1-Q7 in DMSO. Maximum excitation wavelengths for Q1, Q2, Q3, Q4, Q5, Q6 and Q7 were 645, 650, 650, 655, 685, 645 and 645 nm, respectively.

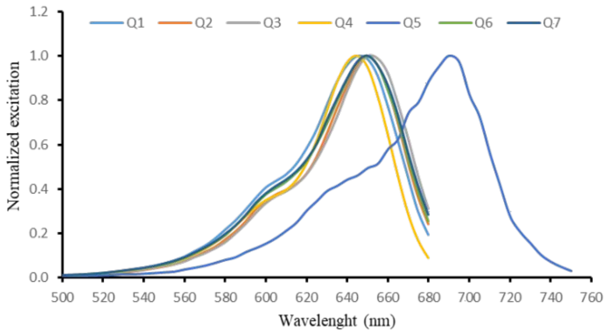

Figure S20. Normalized excitation spectra of Q1-Q7 in H_2O. Maximum excitation wavelengths for Q1, Q2, Q3, Q4, Q5, Q6 and Q7 were 645, 650, 650, 645, 690, 650 and 650 nm, respectively.

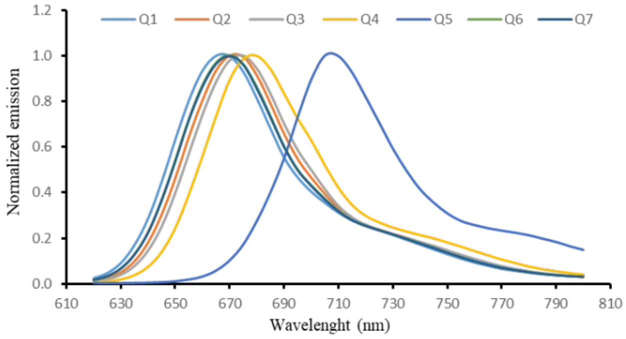

Figure S21. Normalized emission spectra of Q1-Q7 in DMSO. Maximum emmision wavelengths for Q1, Q2, Q3, Q4, Q5, Q6 and Q7 were 670, 670, 675, 680, 705, 670 and 670 nm, respectively.

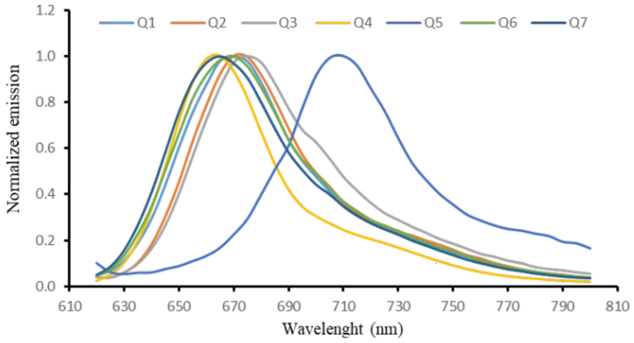

Figure S22. Normalized emission spectra of Q1-Q7 in H_2O. Maximum emmision for Q1, Q2, Q3, Q4, Q5, Q6 and Q7 were 670, 670, 675, 665, 710, 670 and 665 nm, respectively.

.

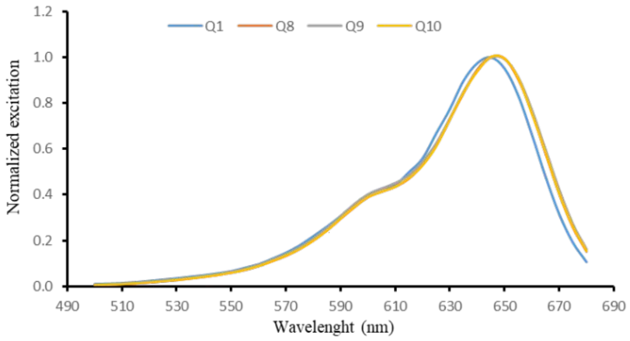

Figure S23. Normalized excitation spectra of Q1 and Q8-Q10 in DMSO. Maximum excitation wavelengths for all were 645 nm.

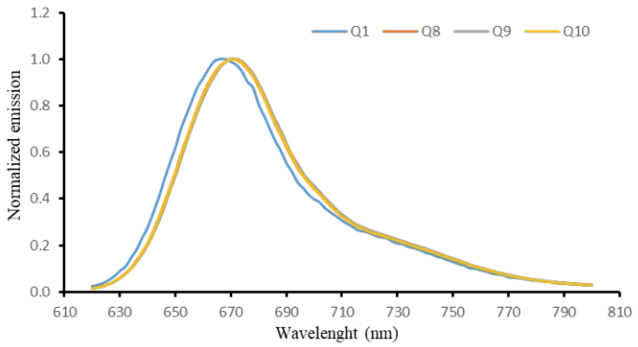

Figure S24. Normalized emission spectra of quenchers Q1 and Q8-Q10 in DMSO. Maximum emmision wavelengths for Q1, Q8, Q9 and Q10 were 668, 670, 670 and 670 nm, respectively.

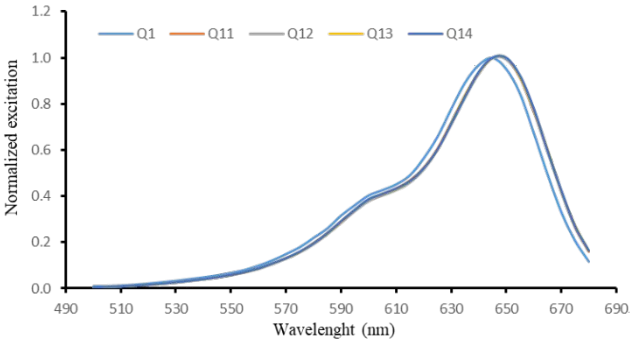

Figure S25. Normalized excitation spectra of quenchers Q1 and Q11-Q14 in DMSO. Maximum excitation wavelengths for all were 645 nm.

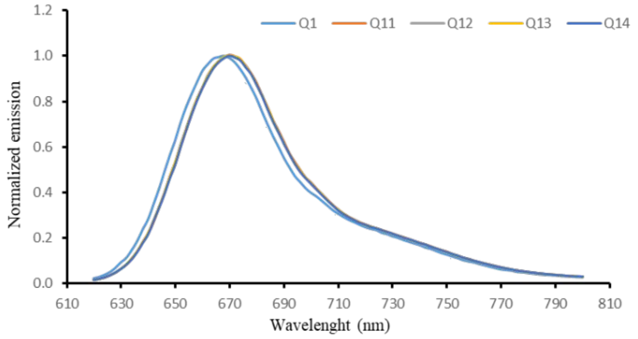

Figure S26. Normalized emission spectra of quenchers 1, 11-14 (Q1, Q11-Q14) in DMSO. Maximum emmision wavelengths for Q1, Q11, Q12, Q13 and Q14 were 668, 668, 670, 668 and 670 nm, respectively.

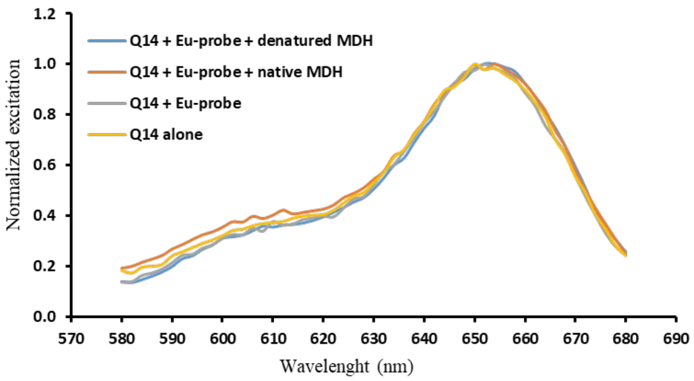

Figure S27. Normalized excitation spectra of Q14 alone, Q14 + Eu-probe, Q14 + Eu-probe + native MDH and Q14 + Eu-probe + denatured MDH at HEPES buffer (10 mM, pH 7.5, 10 mM NaCl, 0.01% Brij 30). Maximum excitation wavelengths for Q14 alone, Q14 + Eu-probe, Q14 + Eu-probe + native MDH and Q14 + Eu-probe + denatured MDH were 650, 652, 654 and 652 nm, respectively.

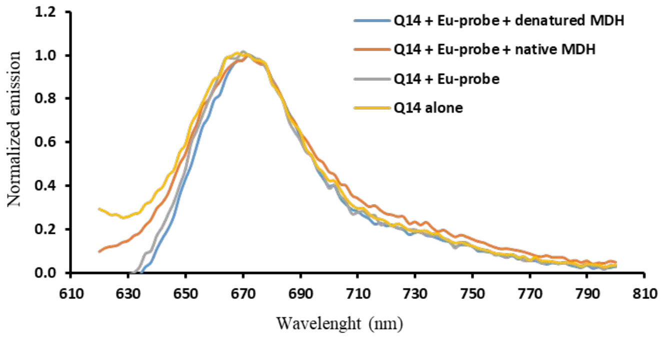

Figure S28. Normalized emission spectra of Q14 alone, Q14 + Eu-probe, Q14 + Eu-probe + native MDH and Q14 + Eu-probe + denatured MDH at HEPES buffer (10 mM, pH 7.5, 10 mM NaCl, 0.01% Brij 30). Maximum emmision wavelengths for Q14 alone, Q14 + Eu-probe, Q14 + Eu-probe + native MDH and Q14 + Eu-probe + denatured MDH were 668, 670, 672 and 672 nm, respectively.

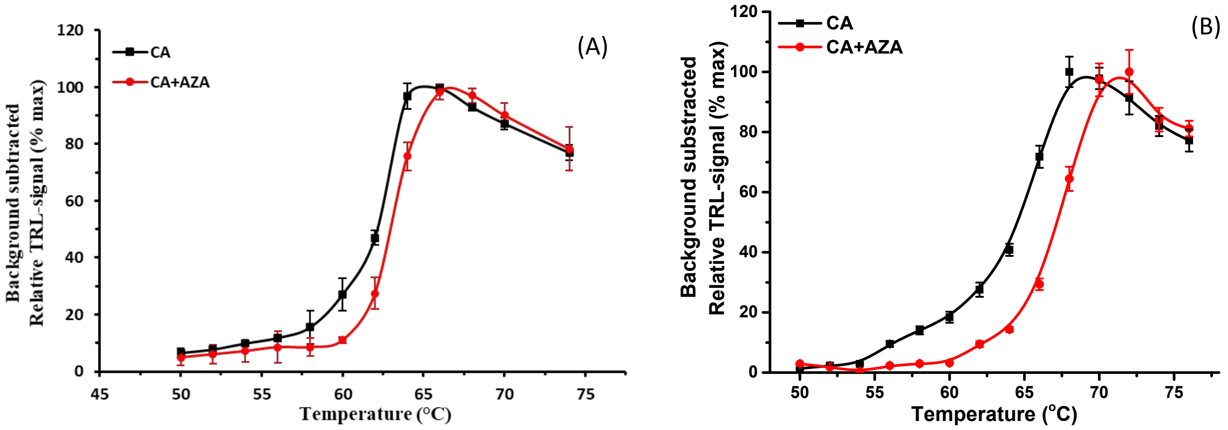

Figure S29. Melting curves of BCA in the absence and presence of its inhibitor acetazolamide (AZA) in one-step Peptide-Probe assays using (A) BCA (33 nM), AZA (0.55 µM), the Eu-probe (1 nM) and Q14 (12.5 nM) at Tris buffer (10 mM, pH 7.5, 0.01% Triton X-100). The T_m values were 61.9 ± 0.7 and 63.4 ± 0.7 °C for BCA and BCA+AZA, respectively, and (B) BCA (300 nM), AZA (3.75 µM), the Eu-probe (1 nM) and Q14 (10 nM) at HEPES buffer (10 mM, pH 7.5, 10 mM NaCl, 0.01% Brij 30). The T_m values were 64.6 ± 0.3 and 67.5 ± 0.4 °C for BCA and BCA+AZA, respectively. Data are expressed as the mean ± SD.

Table S1. Melting temperature (T_m) values of bovine carbonic anhydrase (BCA II) using different methods and conditions.

Entry	$T_{m,}$ (°C)	Method	Condition	Ref
1	64	DSC	0.12-0.20 mg/mL (4-6.6 µM) in 25 mM sodium acetate or, 100 mM NaCl, 0.5 mM EDTA	12
2	71.8	UV @ 280 nm	2 µM in 100 mM Tris buffer, pH 7.5	13
3	64.3	Differential pH	7.1 mg/mL (237 µM) in 0.1 N NaCl.	14
4	64.2	UV @ 280 nm	10.3 µM in 50 mM Tris buffer, pH 7.5	15
5	67	DSC	5-8 µM in 50 mM Tris buffer, pH 7.4	16
6	65.8	CD	5-8 µM in 10 mM sodium phosphate buffer, pH 7.4	16
7	62.5	UV @ 291 nm	16 µM in 0.09 M Tris buffer, pH 7.55	17
8	68.2	DSF	10 µM in 10 mM NaH_2PO_4/Na_2HPO_4, pH 7.0, 10 mM NaCl	18
9	70.9	DSC	10-15 µM in 10 mm phosphate buffer, pH 8	19

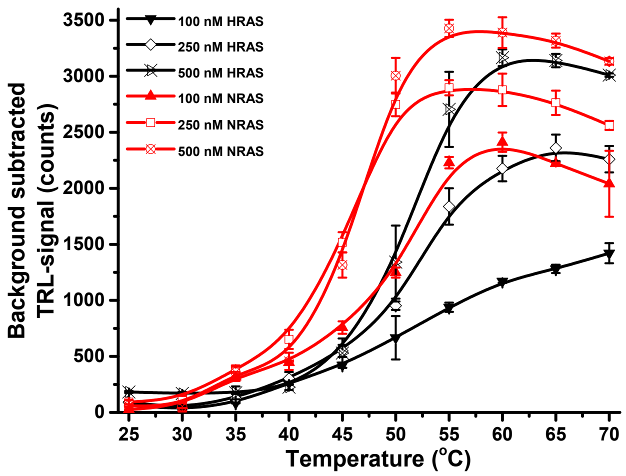

Figure S30. Melting curves of HRAS and NRAS at different concentrations (100, 250, 500 nM) in one-step Peptide-Probe assays using the Eu-probe (1 nM) and Q14 (10 nM) at HEPES buffer (10 mM, pH 7.5, 10 mM NaCl, 0.01% Brij 30). T_m values were 48.9 ± 2, 49.4 ± 0.5, 50.3 ± 6, 47.3 ± 0.5, 46.5 ± 0.6 and 46.4 ± 1.2 °C for HRAS (100 nM), HRAS (200 nM), HRAS (500 nM), NRAS (100 nM), NRAS (200 nM) and NRAS (500 nM), respectively. Data are expressed as the mean ± SD.

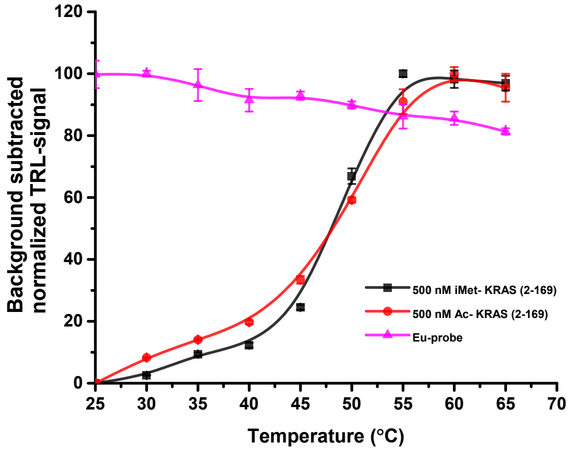

Figure S31. Melting curves of Ac-KRAS (2-169) and iMet-KRAS (2-169) (each 500 nM) in one-step Peptide-Probe assays using the Eu-probe (1 nM) and Q14 (10 nM) at HEPES buffer (10 mM, pH 7.5, 10 mM NaCl, 0.01% Brij 30). The T_m values were 46.6 ± 1 and 48.4 ± 1.5 °C for Ac-KRAS (2-169) and iMet-KRAS (2-169), respectively. Data are expressed as the mean ± SD.

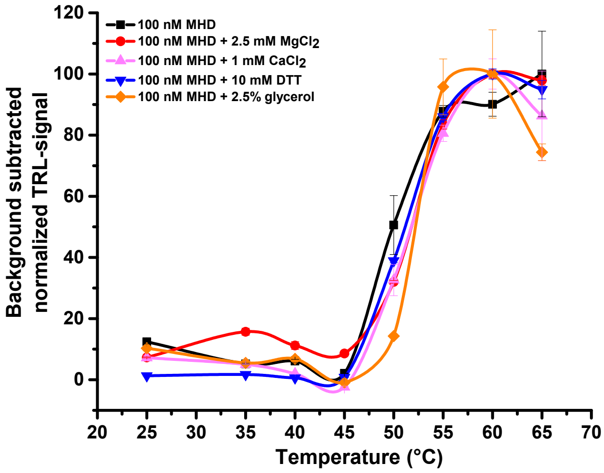

Figure S32. Melting curves of MDH (100 nM) in both the absence and presence of $MgCl_2$ (2.5 mM), $CaCl_2$ (1 mM), dithiothreitol (DTT, 10 mM) and glycerol (2.5 %) using the one-step Peptide-Probe assays with the Eu-probe (1 nM) and Q14 (10 nM) at HEPES buffer (10 mM, pH 7.5, 10 mM NaCl, 0.01% Brij 30). The T_m values were 50 ± 0.3, 52.2 ± 0.5, 51.9 ± 0.7, 51 ± 0.3 and 52 ± 0.7 °C for MDH (100 nM), MDH (100 nM) + $MgCl_2$ (2.5 mM), MDH (100 nM) + $CaCl_2$ (1 mM), MDH (100 nM) + DTT (10 mM) and MDH (100 nM) + glycerol (2.5 %), respectively.

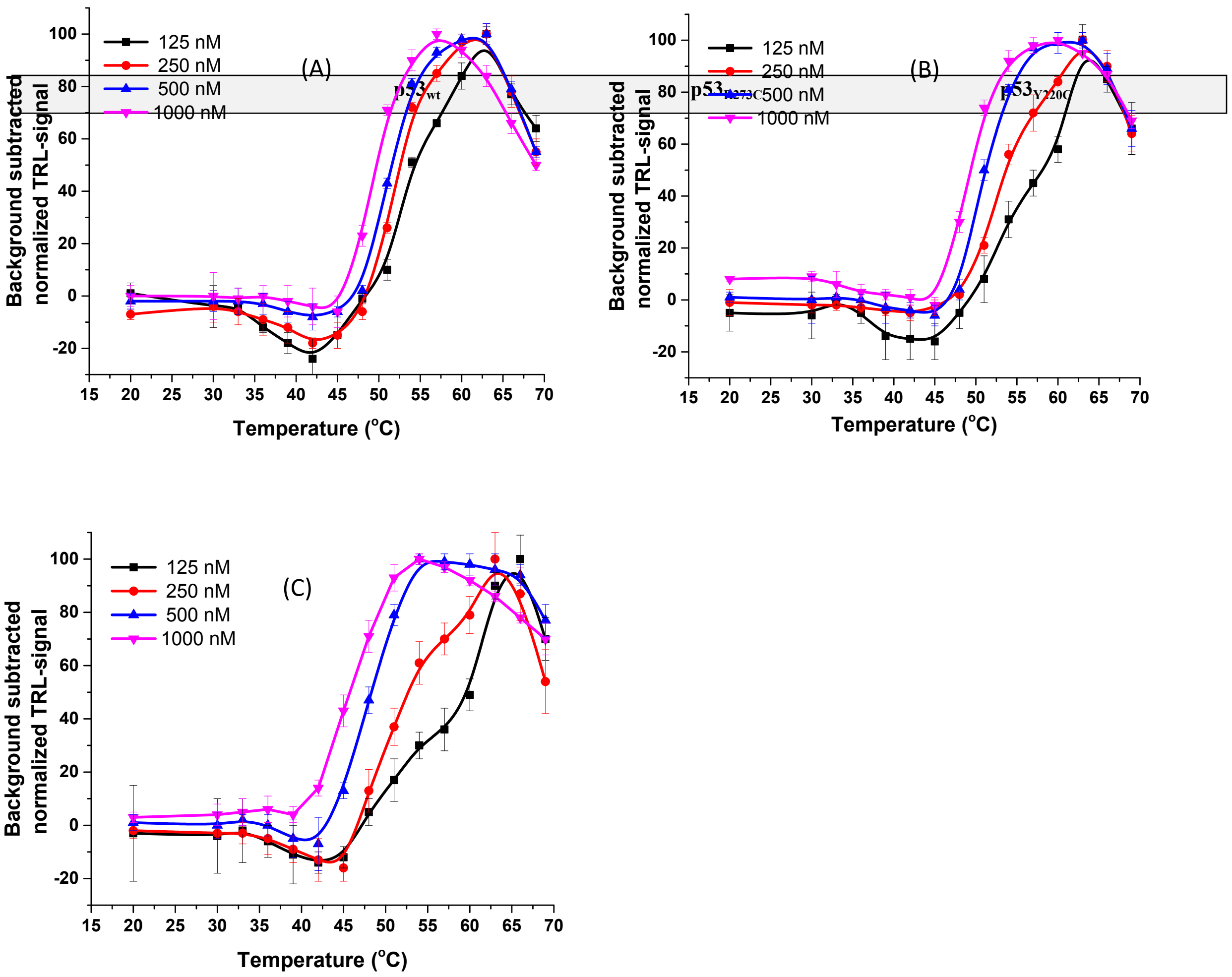

Figure S33. Melting curves of (A) p53$_{wt}$, (B) p53$_{R273C}$ and (C) p53$_{Y220C}$ at different concentrations (125, 250, 500, 1000 nM) in the one-step Peptide-Probe assays using the Eu-probe (6 nM) and Q14 (15 nM) in HEPES buffer (10 mM, pH 7.4, 30 mM NaCl, 0.01% Triton X-100). At high protein concentration, the profiles are clearly one-phasic while at lower concentrations the profiles are two-phasic indicating fine-structural melting profiling. It is also obvious from the measured data that more fine-structural information is obtained at reduced protein concentration. Within the aims of the current study, we did not further investigate this. However, this may originate from dimeric structure of the protein as the dimer dissociation constant is at the low nanomolar range indicating that the proteins were as dimers at all measured concentrations.[20] Moreover, p53$_{Y220C}$ had a lower T_m value and higher ΔT_m (phase 2 vs. phase 1) compared to the other p53 proteins, which may reflect the effect of this specific mutation on protein stability and structural properties.[21] The biphasic data are interesting as all current methodologies measure thermal profiles at elevated concentrations above 1000 nM concentration – apparently missing some fine-structural information leading to concentration-driven T_m value data interpretation.

		T_m (°C) [a]			T_m (°C) [a]			T_m (°C) [a]		
**Method**	**Conc (nM)**	**Phase 1**	**Phase 2**	**S/B**	**Phase 1**	**Phase 2**	**S/B**	**Phase 1**	**Phase 2**	**S/B**
Peptide-Probe	125 [b]	52.5 ± 1.4	55.1 ± 0.9	5.4	52.8 ± 0.8	59.6 ± 1.9	5.2	49.7 ± 0.7	59.8 ± 0.1	6.4
Peptide-Probe	250 [b]	51.9 ± 0.2		6.6	52.3 ± 0.2	54.8 ± 0.5	6.6	49.9 ± 0.7	55.5 ± 0.9	6.7
Peptide-Probe	500	51.3 ± 0.2		8.2	51.1 ± 0.2		7.0	48.0 ± 0.3		7.1
Peptide-Probe	1000	49.6 ± 0.2		9.7	49.3 ± 0.2		7.6	46.0 ± 0.2		7.7
DSF	2000	47.1 ± 0.1		8.5	46.6 ± 0.1		6.1	38.6 ± 0.1		8.1

Table S2. Comparison of Thermal Profile Data for $p53_{wt}$, $p53_{R273C}$, and $p53_{Y220C}$ Using the One-Step Peptide-Probe and DSF Methods.

[a] The T_m values calculated with the DSF method were lower than those obtained with the one-step Peptide-Probe method.
[b] Two-phasic curves were obtained with the one-step Peptide-Probe method.

References

(1) Kopra, K.; Vuorinen, E.; Abreu-Blanco, M.; Wang, Q.; Eskonen, V.; Gillette, W.; Pulliainen, A. T.; Holderfield, M.; Härmä, H. Homogeneous Dual-Parametric-Coupled Assay for Simultaneous Nucleotide Exchange and KRAS/RAF-RBD Interaction Monitoring. *Anal. Chem.* **2020**, 92, 4971−4979.

(2) Kopra, K.; Valtonen, S.; Mahran, R.; Kapp, J. N.; Hassan, N.; Gillette, W.; Dennis, B.; Li, L.; Westover, K. D.; Plückthun, A.; Härmä. H. Thermal Shift Assay for Small GTPase Stability Screening: Evaluation and Suitability. *Int. J. Mol. Sci.* **2022**, 23, 7095.

(3) Dharmaiah, S.; Tran, T. H.; Messing, S.; Agamasu, C.; Gillette. W. K.; Yan, W.; Waybleft, T.; Alexander, P.; Esposito, D.; Nissley, D. V.; McCormick, F.; Stephen, A. G.; Simanshu, D. K. Structures of N-Terminally Processed KRAS Provide Insight into the Role of N-Acetylation. *Sci. Rep.* **2009**, 9, 10512.

(4) Valtonen, S.; Vuorinen, E.; Kariniemi, T.; Eskonen, V.; Quesne, J. L.; Bushell, M.; Härmä, H.; Kopra, K. Nanomolar Protein−Protein Interaction Monitoring with a Label-Free Protein-Probe Technique. *Anal. Chem.* **2020**, 92, 15781–15788.

(5) Wilczynska, A.; Gillen, S. L.; Schmidt, T.; Meijer, H. A.; Jukes-Jones, R.; Langlais, C.; Kopra, K.; Lu, W.; Godfrey, J. D.; Hawley, B. R.; Hodge, K.; Zanivan, S.; Cain, K.; Quesne, J. L.; Bushell, M. eIF4A2 Drives Repression of Translation at Initiation by Ccr4-Not Through Purine-Rich Motifs in the 5′UTR. *Genome Biol.* **2019**, 20, 262.

(6) Ashok, Y.; Miettinen, M.; Kimio Hirabae de Oliveira, D.; Tamirat, M. Z.; Näreoja, K.; Tiwari, A.; Hottiger, M. O.; Johnson, M. S.; Lehtio, L.; Pulliainen, A. P. Discovery of Compounds Inhibiting the ADP-Ribosyltransferase Activity of Pertussis Toxin. *ACS Infect. Dis.* **2020**, 6, 588−602.

(7) Owens, E. A.; Bruschi, N.; Tawney, J. G.; Henary, M. A Microwave-Assisted and Environmentally Benign Approach to the Synthesis of Near-Infrared Fluorescent Pentamethine Cyanine Dyes. *Dyes Pigm.* **2015**, 113, 27-37.

(8) Barbero, N.; Magistris, C.; Park, J.; Saccone, D.; Quagliotto, P.; Buscaino, R.; Medana, C.; Barolo, C.; Viscardi, G. Microwave-Assisted Synthesis of Near-Infrared Fluorescent Indole-Based Squaraines. *Org. Lett.* **2015**, 17, 3306−3309.

(9) Winstead, A. J.; Fleming, N.; Hart, K.; Toney, D. Microwave Synthesis of Quaternary Ammonium Salts. *Molecules* **2008***, 13,* 2107-2113.

(10) Winstead, A. J.; Nyambura, G.; Matthews, R.; Toney, D.; Oyaghire, S. Synthesis of Quaternary Heterocyclic Salts. *Molecules* **2013**, *18*, 14306-14319.

(11) Beckford, G.; Owens, E.; Henary, M.; Patonay, G. The Solvatochromic Effects of Side Chain Substitution on the Binding Interaction of Novel Tricarbocyanine Dyes with Human Serum Albumin. *Talanta* **2012**, 92, 45-52.

(12) Matulis, D.; Kranz, K. J.; Raymond Salemme, F.; Todd, J. M. Thermodynamic Stability of Carbonic Anhydrase: Measurements of Binding Affinity and Stoichiometry Using ThermoFluor. *Biochemistry* **2005,** *44,* 5258-5266.

(13) Safarian, S.; Bagheri, F.; Moosavi-Movahedi, A. A.; Amanlou, M.; Sheibani, N. Competitive Inhibitory Effects of Acetazolamide upon Interactions with Bovine Carbonic Anhydrase II. *Protein J.* **2007**, 26, 371-385.

(14) McCoy, L. F., Jr.; Wong, K.-P. Renaturation of Bovine Erythrocyte Carbonic Anhydrase B Denatured by Acid, Heat, and Detergent. *Biochemistry* **1981**, *20*, 3062-3067.

(15) Sarraf, N.; Saboury, A.; Ranjbar, B.; Moosavi-Movahedi, A. Structural and Functional Changes of Bovine Carbonic Anhydrase as a Consequence of Temperature. *Acta Biochim. Pol.* **2004**, 51, 665–671.

(16) Lisi, P. G.;Hughes, P. R.; Wilcox, E. D. Coordination Contributions to Protein Stability in Metal‑Substituted Carbonic Anhydrase. *J Biol Inorg Chem*. **2016**, 21, 659–667.

(17) Lavecchia. R.; Zugaro, M. Thermal Denaturation of Erythrocyte Carbonic Anhydrase. **1991,** 292, 162-164.

(18) Krasavin, M.; Kalinin, S.; Zozulya, S.; Gryniukova, A.; Borysko, P.; Angeli, A.; Supuran, T. C. Screening of Benzenesulfonamide in Combination with Chemically Diverse Fragments Against Carbonic Anhydrase by Differential Scanning Fluorimetry, *J. Enzyme Inhib. Med. Chem*. **2020**, 35:1, 306-310.

(19) Gitlin, I.; Gudiksen, L. K.; Whitesides, M. G. Effects of Surface Charge on Denaturation of Bovine Carbonic Anhydrase. *ChemBioChem* **2006**, 7, 1241-1250.

(20) Rajagopalan, S.; Huang, F.; Fersht, A. R. Single-Molecule Characterization of Oligomerization Kinetics and Equilibria of the Tumor Suppressor p53. *Nucleic Acids Res*. **2011**, 39, 2294-2303.

(21) Kulandaisamy, A.; Zaucha, J.; Frishman, D.; Gromiha, M. M. MPTherm-pred: Analysis and Prediction of Thermal Stability Changes upon Mutations in Transmembrane Proteins. *J. Mol. Biol.* **2021**, 433, 166646.
